# A Meta-Analysis of Biostimulant Yield Effectiveness in Field Trials

**DOI:** 10.3389/fpls.2022.836702

**Published:** 2022-04-14

**Authors:** Jing Li, Thijs Van Gerrewey, Danny Geelen

**Affiliations:** HortiCell, Department of Plants and Crops, Faculty of Bioscience Engineering, Ghent University, Ghent, Belgium

**Keywords:** meta-analysis, biostimulant, crop yield, sustainable agriculture, open-field trial, climate, soil quality

## Abstract

Today's agriculture faces many concerns in maintaining crop yield while adapting to climate change and transitioning to more sustainable cultivation practices. The application of plant biostimulants (PBs) is one of the methods that step forward to address these challenges. The advantages of PBs have been reported numerous times. Yet, there is a general lack of quantitative assessment of the overall impact of PBs on crop production. Here we report a comprehensive meta-analysis on biostimulants (focus on non-microbial PBs) of over one thousand pairs of open-field data in a total of 180 qualified studies worldwide. Yield gains in open-field cultivation upon biostimulant application were compared across different parameters: biostimulant category, application method, crop species, climate condition, and soil property. The overall results showed that (1) the add-on yield benefit among all biostimulant categories is on average 17.9% and reached the highest potential via soil treatment; (2) biostimulant applied in arid climates and vegetable cultivation had the highest impact on crop yield; and (3) biostimulants were more efficient in low soil organic matter content, non-neutral, saline, nutrient-insufficient, and sandy soils. This systematic review provides general biostimulant application guidelines and gives consultants and growers insights into achieving an optimal benefit from biostimulant application.

## Introduction

By 2050, the risk of hunger is predicted to rise by 30% due to climate change and the expected population increase (Van Dijk et al., 2021). To meet future food production requirements, the impact of climate change on crop production needs to be addressed. How we will achieve this ambitious goal is currently debated. Various conventional (e.g., fertilization) and novel bioengineering strategies (e.g., genetically modified crops) are extensively developed to boost crop production and ensure food security and safety (Bailey-Serres et al., 2019). As a general consensus is emerging synthetic fertilizers that cause environmental threats to the local and global ecosystems (Koli et al., 2019), plant biostimulants (PBs) are potentially a tool to mitigate climate change-induced stress and reduce the dependency on chemical fertilizers (Hunter et al., 2017). The European Commission aims to replace 30% of chemical fertilizers with bio-based alternatives by 2050 (Hansen, 2018). The application of PBs, is a more sustainable agricultural practice to preserve crop yield under reduced fertilizer conditions (Gupta et al., 2020). The global market value of PBs is expected to reach USD 3 billion in 2021, with a cumulative annual growth rate (CAGR) of about 13% until 2025 (EBIC, 2021). Nevertheless, there is no clear view of how efficient PBs really are.

The European Commission has categorized PBs under the framework of fertilizing products [Regulation (Eu) 2019/1009, 2019]. Briefly, it states that PBs are products that stimulate plant growth and improve one or more additional functions: nutrient use efficiency, abiotic stress tolerance, crop quality traits, and availability of confined nutrients in the soil or plant rhizosphere. Furthermore, Regulation (EU) 2019/1009 cataloged two distinct categories based on whether the stimulatory bioactivity is of microbial or non-microbial origin, and this may require an even more refined classification (Rouphael and Colla, 2020). Within the most accepted subcategories, microbial PBs consist of arbuscular mycorrhizal fungi (AMF) and plant growth-promoting rhizobacteria (PGPR) (Rouphael and Colla, 2020). Commonly, 6 subcategories of non-microbial PBs are distinguished: chitosan (Chi), humic and fulvic acids (HFA), animal and vegetal protein hydrolysates (PHs), phosphites (Phi), seaweed extracts (SWE), and silicon (Si). More recently, an additional group of PBs that have received much attention are the plant extract-based PBs (PE) (excluding SWE) and are included as a separate class of PBs (Du Jardin, 2015; Bio4Safe, 2021). Aside from these complex mixtures of PBs, products with a single active compound are not included in this review because we consider that the majority of PBs are complex mixtures (Du Jardin, 2015; García-García et al., 2020). The European Biostimulant Industry Council (EBIC) proposed several general principles to regulate and justify the claims made by manufacturers with regards to PBs efficiency (Ricci et al., 2019). A European legal framework for PBs regarding standardization of sampling, denominations, marking, and test methods are currently under development and will be fully released in 2024 (CEN Technical Committees, 2021). Therefore, sufficient high-quality and credible experimental data is required from PBs producers to support the claims of PBs products and provide valuable practical advice for the users. Recently, a biostimulant database was launched for growers, gathering PBs product information, plant trails, and scientific data on crop quality, water nutrient use efficiency, and stress tolerance (Bio4Safe, 2021). However, an overall in-depth evaluation of PBs performance is still missing.

Crop yield enhancement is a popular claim listed in the product description of many PBs (Ricci et al., 2019). As various environmental factors and management practices influence yield performance (Liliane and Charles, 2020), empirical knowledge that depends on different experimental conditions is of critical value for the farmer. Because of the variability in agronomic management and environmental conditions, studies with similar or identical PBs have resulted in different effectiveness data (Schütz et al., 2018). As crop yield is a multi-trait property, meta-analysis has been conducted to gain insight into the impact of soil property (Oldfield et al., 2019), climate change (Challinor et al., 2014), and microbial PBs application (Schütz et al., 2018). Hence, effectiveness remains poorly understood to what extent these variables affect non-microbial PBs (designated as biostimulants in later text).

In this study, we performed a meta-analysis to (1) estimate crop yield improvement by biostimulants, (2) understand the relationship between crop yield and biostimulant application method, and (3) assess the impact of environmental variables on the performance of biostimulants in open-field cultivation.

## Materials and Methods

### Literature Review

Thomson Reuters' Web of Science, Elsevier's Scopus, and Google Scholar were queried until July 2021 for peer-reviewed publications identified using the keywords “biostimulant AND crop AND (yield OR biomass)”. Additional studies were also selected based on citations occurring in the selected papers and relevant reviews. Studies were selected for the analyses using five criteria: (1) crop yield data were obtained from open-field trials or walk-in tunnels (open on both sides), excluding pot and greenhouse experiments; (2) marketable crop yield was reported as it represents traceable agro-economic value; (3) the studies contained pairwise comparisons between single biostimulant-treated and corresponding non-treated control plants, using the same application method under the same geo-climatic and crop management; (4) the yield means, their standard deviation (SD), and the number of replications were provided separately; (5) the studies were written in English and available in full text. A total of 1,108 paired observations from 181 empirical studies (Supplementary Data 1) were identified after two rounds of screening of titles, abstracts, and full texts analyzed in this study following the PRISMA-P statement (Supplementary Figure 1) (Page et al., 2021).

### Data Collection

Experimental crop yield data were collected from the original tables or extracted from the attached figures using WebPlotDigitizer (Rohatgi, 2020). The methodology section obtained other information, including the field site location, crop species, soil properties, biostimulant product information, application method, dose, frequency, and whether interannual studies were performed. All data were compiled in one dataset (Supplementary Data 2) after conversion to uniform metrics for each variable.

#### Moderator Variables

Four main groups of moderator variables were considered to investigate further potential crop yield effectors, including experimental-, plant-, climate-, and soil-related parameters. Table 1 shows the classifications of all categorical moderators.

**Table 1:** The description of classifications involved in categorical moderators.

**Categorical moderators**	**Classifications involved**
Climate categories^a^	A (Af, Am, As, Aw); B (BWk, BWh, BSk, BSh); C (Cfa, Cfb, Csa, Csb, Cwa); D (Dfa, Dfb, Dfc, Dsa).
Crop species in categories	Cereals (wheat, maize, oat, barley, rice, quinoa); Fruits (including nuts) (grape, mango, apricot, cherry, plum, mandarin, blueberry, apple, strawberry, pear, pistachio nut, papaya, citrus, sugarcane); Legumes (soybean, faba bean, black gram, common bean, pea, cowpea, mung bean, snap bean); Others (fennel, berseem clover, cardoon, dragonhead, geranium, sesame, lemon, hyssop, grass mixture, ryegrass, timothy, alfalfa, cotton, basil, honeysuckle, rape, vetch, chamomile, olive, zinnia, sugar beet, niger, milk thistle, meadow, mint, red clover); Root/tuber crops (potato); Vegetables (eggplant, rocket, tomato, okra, sweet pepper, onion, pepper, lettuce, garlic, broccoli, carrot, endive, cabbage, spinach, cucumber, celery).
Degree of soil pH	Strongly acid (5.1–5.5); Moderately acid (5.6–6.0); Slightly acid (6.1–6.5); Neutral (6.6–7.3); Moderately alkaline (7.9–8.4); Strongly alkaline (8.5–9.0).
Degree of soil salinity by ECe (dS/m)	Nonsaline (0–2.0); Slightly saline (2.1–4.0); Moderately saline (4.1–8.0); Strongly saline (>8.1).
Soil P Levels (ppm)	Very low (<16); Low (16–25); Medium (26–35); Optimal (>36).
Soil K levels (ppm)	Very low (<61); Low (61–60); Medium (91–130); Optimal (>131).

a *According to Köppen-Geiger climate classification (Peel et al., 2007) on main climates, A, equatorial; B, arid; C, warm temperate; D, snow; on precipitation: W, desert; S, steppe; f, fully humid; s, summer dry; w, winter dry; m, monsoonal; on temperature: h, hot arid; k, cold arid; a, hot summer; b, warm summer; c, cool summer*.

##### Biostimulant Categories and Methods of Application

The classification of biostimulants was based on the main bioactive substances: Chi, HFA, PHs, Phi, SWE, Si, and PE (Du Jardin, 2015; Rouphael and Colla, 2020). Detailed information about the available natural resources and the major bioactive compounds of these biostimulants are shown in Supplementary Table 1. Moringa leaf extract (MLE) was separated as a subgroup of interest, and the rest were other PE under the PE group.

The application methods are specified as foliar, soil, and seed treatment. Direct biostimulant application in the soil and introduction *via* irrigation water were considered soil treatments. Biostimulant application frequency indicates the total number of foliar applications, where “0” indicates continuous treatment with a specific time interval. The biostimulant application dose was only defined within dose-response studies, where “1” was used for the highest biostimulant concentration, “0” for non-treated conditions, and the other doses were expressed as corresponding relative concentrations. For interannual studies, annual crop cultivation was labeled as “0” while the rest of continuous crop production was marked as the order of successive years.

##### Crop Categories and Environmental Parameters

Cultivated crop species were grouped into 6 main crop categories (cereals, vegetables, fruits, legumes, root/tuber crops, and other crops) following the Food and Agriculture Organization of the United Nations (FAO) classification of agricultural crops (FAO, 2005). In addition, sugar crops (sugar cane and sugar beet), medicinal plants (e.g., cardoon, zinnia, and basil), oilseed crops (rapeseed and olive), grasses (e.g., alfalfa, ryegrass, and timothy), spice crops (cinnamon and fennel), and fiber crops (cotton) were added to the “other crops” class due to the limited number of relevant studies.

The locations, including city and country names, were converted to latitude and longitude with decimal degree coordinates. Next, the climate zone was categorized according to the Köppen-Geiger climate classification (Peel et al., 2007) using R software version 4.0.2 (R Core Team, 2020) equipped with package “kgc” version 1.0.0.2 (Bryant et al., 2017). Finally, the geographical map of the identified studies was visualized using ArcMap (Esri, 2020). Four main climates (equatorial, arid, warm temperate, and boreal) and six subclasses (desert, steppe, monsoonal, summer dry, winter dry, and fully humid) are determined by vegetation and temperature and precipitation, were covered in this study. As regular irrigation was commonly applied during cultivation, studies with artificial drought stress experiments were excluded.

For soil physicochemical properties, soil texture was assigned to 12 classes according to the fractions of clay, silt, and sand particles in the topsoil (0–30 cm), as described by the soil texture triangle (Soil Science Division Staff, 1993). Soil acidity and alkalinity, a measure for soil reaction, were expressed as soil pH measured in water or converted in CaCl2 or KCl (Land Resources Management Unit Commission, 2010). The soil pH levels ranged from 3.5 to 8.4 and were split into six groups (strongly acid, moderately acid, slightly acid, neutral, moderately alkaline, and strongly alkaline) triangle (Soil Science Division Staff, 1993). Electrical conductivity (EC) of soil standard saturated paste extract (ECe), a measure for soil salinity, was represented as the standard EC, and EC measured in soil-water extracts were converted (Kargas et al., 2018). The degree of soil salinity was allocated into five levels (non-saline, slightly saline, moderately saline, and strongly saline) for all soils (Smith and Doran, 1997). The soil organic matter (SOM) was considered a critical indicator of soil health (Soil Health Institute, 2018). SOM was transposed from available soil organic carbon (SOC), which assumedly contributed to 58% of the mass of SOM (Edwards, 2021). Soil total nitrogen (N) indicates the percentage of organic and inorganic N forms (Marx et al., 1996). Furthermore, soil available nutrients for plant uptake are associated with three macronutrients, N, phosphorus (P), and potassium (K) content (ppm concentrations), where soil available N content (ppm) only includes plant-available nitrate- and ammonium-N (Horneck et al., 2011). For easier understanding in agronomy guide, soil fertility was interpreted to four levels (very low, low, medium, and optimal) based on available P and K concentrations and the expected yield potential without fertilization for most agronomic crops (Snyder et al., 1993).

#### Missing Values

If the studies only provided the dry biomass, the fresh weight was computed according to the calculated water contents (Spungen, 2005). When outcome errors were unavailable in the original text, the SD was estimated in percentage based on the mean SD of the existing studies per crop category (Schütz et al., 2018). The estimated SD for cereals was 4.25%, legumes 13.4%, root/tuber crops 9.51%, vegetables 9.67%, fruits 16.5%, and for other crops (grasses and medical plants 8.84%, sugar crops 1.55%, oilseed crops 8.92%). Thus, more importantly, the availability of complete raw datasets combined with other descriptive records of trail-related conditions is critical for proper statistical analysis and justifying biostimulant claims of manufacturers (Ricci et al., 2019).

### Data Analysis

The primary outcome of crop yield was defined as the fresh weight (kg·m^−2^) of marketable product, in the case of cereals, the seed or grain yield, shoot biomass for most vegetables or other crops, fruit yield for fruits, and tuber yield for root/tuber crops. The yield response (%) to biostimulant application was calculated using the Equation (1),


(1) $$\begin{array}{r} Yield\;response\;\left(\%\right)=\;\frac{{Yield}_{with\;biostimulant}\;}{{Yield}_{without\;biostimulant}}\;\times 100\% \end{array}$$

where “Yield_with biostimulant_” is the crop yield after treatment with a single biostimulant product and “Yield_without biostimulant_” is the crop yield under non-treated control conditions. A meta-analysis was conducted in R with package “metafor” version 3.0-2 (Viechtbauer, 2010). First, the effect size was calculated with log-transformed ratios of the means and confidence intervals (CI) of each means, with yield response as the main outcome. The variables were assigned according to the major influencing factors of crop production. Next, random-effect models (RE) were fitted with categorical moderators using the restricted maximum-likelihood (REML) estimator method. For categorical moderators, forest plots were applied to visualize the meta-analyses. On the other hand, linear meta-regression analysis on continuous variables was conducted by fitting mixed-effect models (ME). The ME performance was evaluated by *R*^2^ for variance explained (Nakagawa and Schielzeth, 2013). We also investigated the between-study heterogeneity by determining *I*^2^ of each model (Inthout et al., 2016). To further explore the causes of heterogeneity, influence diagnostics was applied to remove outlier cases with large DFBETAS (indicating the change of SDs after study exclusion from model fitting). As a result, twenty-one of the 1,108 studies were removed. Additionally, to eliminate the systematic publication bias in meta-analysis, we evaluated the comprehensiveness of collected data by assessing the asymmetry of the funnel plot with the “regtest” function. Since the asymmetry test was not significant (*p* > 0.05) (Supplementary Figure 2), we thus addressed no issues on publication bias in our dataset and included all the data for this study.

## Results

**Figure 1:**
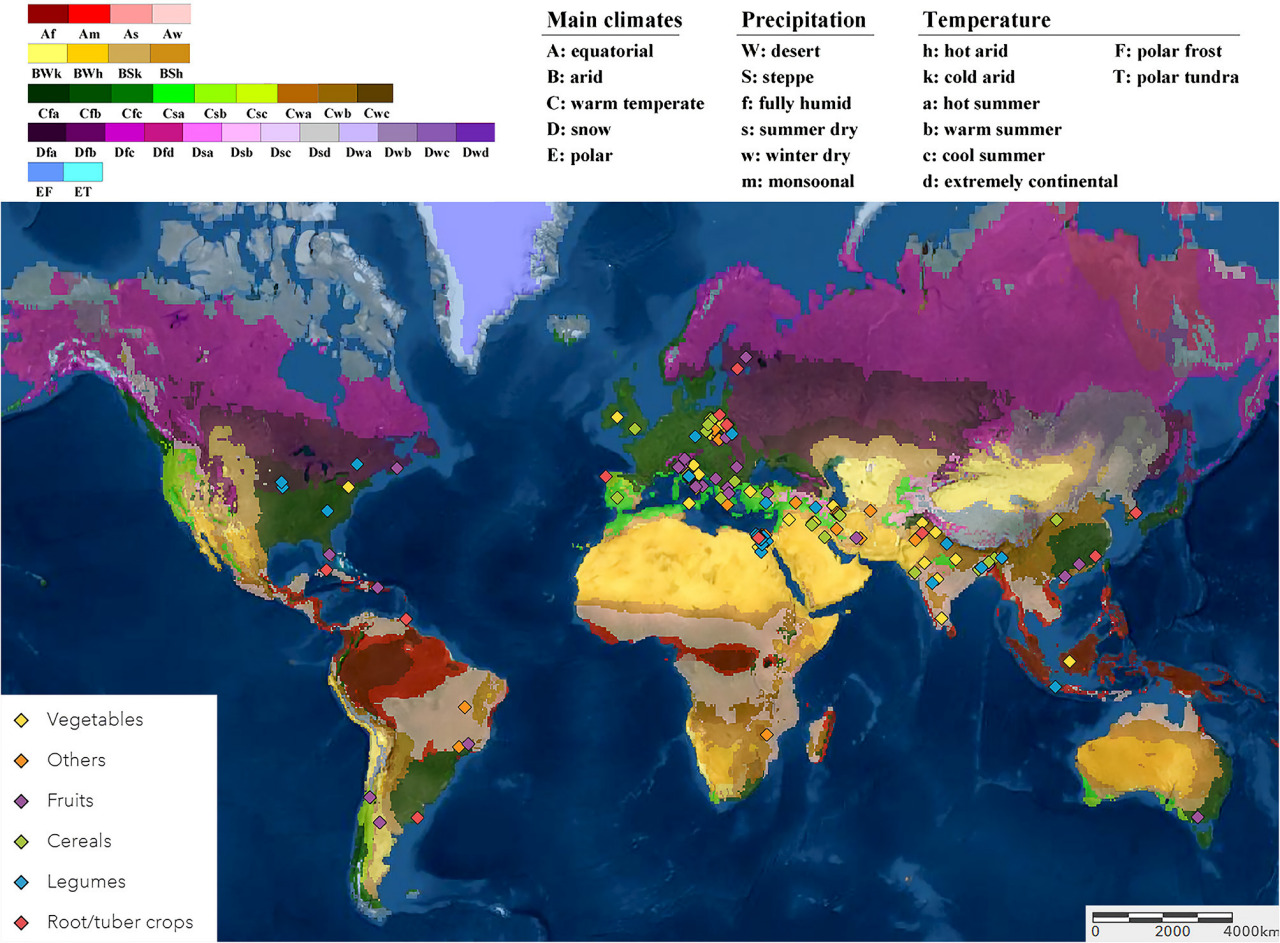
The locations of the open-field studies included in the meta-analysis as displayed on the Köppen-Geiger climate classification (Peel et al., 2007) on the world map (Esri, 2009). The studies were grouped based on the six crop categories of cultivation (cereals, legumes, vegetables, fruits, root/tuber crops, and other crops).

Following a literature survey and selection using the quality criteria described previously, 1,087 paired observations in 180 studies were retained for data extraction and comparison. Most of these studies were performed in the Eurasian and Mediterranean regions under arid and warm temperate climates. The smaller subsection of studies was from the Americas and Southeast Asia, and a single study was conducted in Southern Africa and one in Australia (Figure 1).

### PE Is the Most Efficient Biostimulant

**Figure 2:**
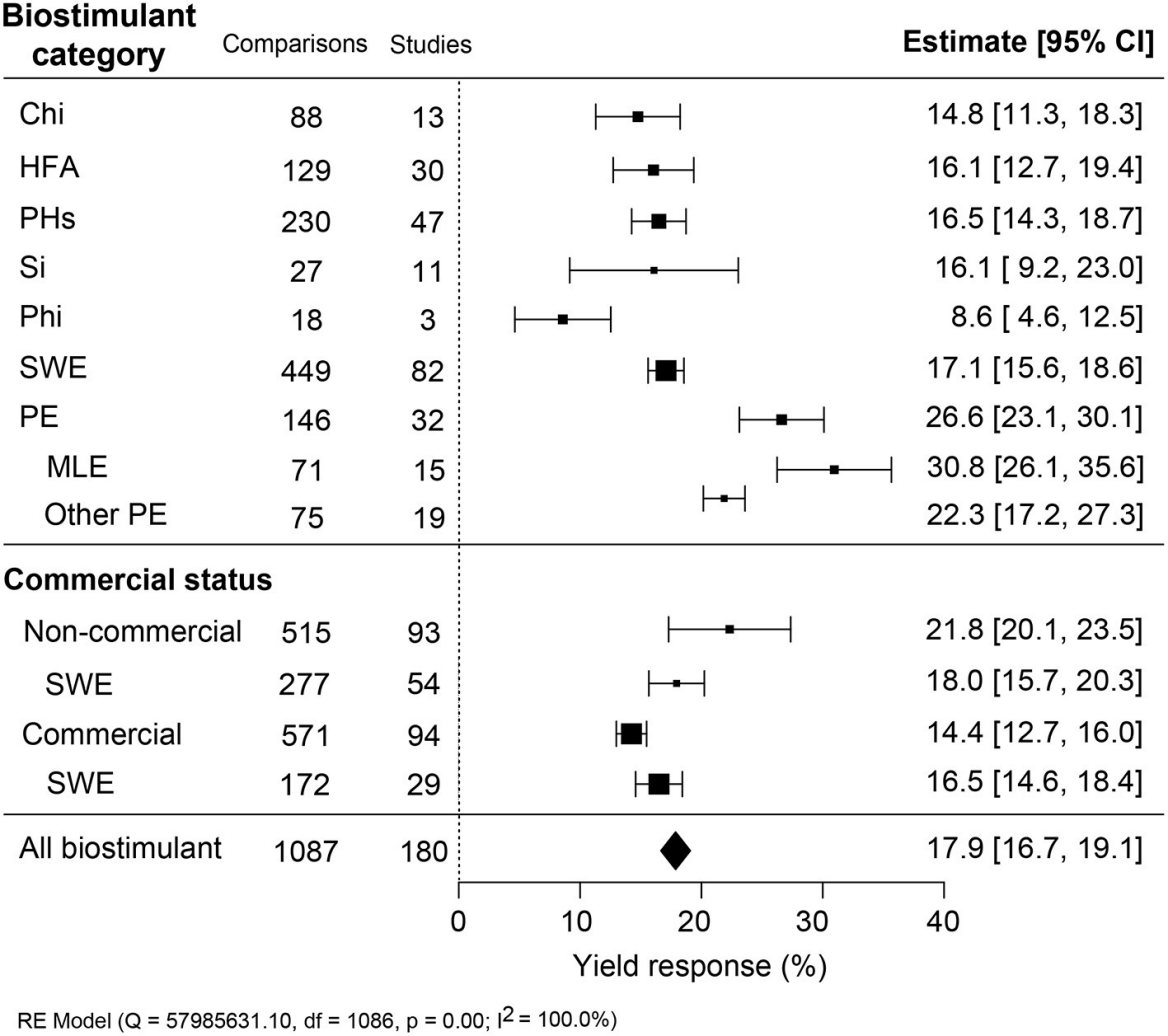
Percentage yield response to biostimulant application affected by the biostimulant category and the commercial status of biostimulant products. The point size correlates to the estimate's precision, and the error bars represent 95% confidence intervals (CI) of mean estimated effect sizes. The number of comparisons and studies is indicated in each line. The combined effect estimates and the heterogeneity test on the random-effect model (RE) are summarized at the bottom, where the heterogeneity test is significant (*p* < 0.001) and *I*^2^ ≥ 75% implies substantial heterogeneity. Chi, Chitosan; HFA, humic and fulvic acids; PHs, protein hydrolysates; Si, silicons; Phi, phosphite; SWE, seaweed extracts; PE, plant extracts; MLE, moringa leaf extract.

The average yield increases induced by reported biostimulant applications in open-field varied between 8.5 and 30.8% between the category (Figure 2). The overall average in crop yield response was 17.9% (CI 16.7–19.0%). The best performing category was PE, with a yield increase of 26.6% (CI 23.1–30.1%). Amongst MLE showed the highest improvement (+30.8%; CI 26.1–35.6%) while other PE (+22.3%; CI 17.2–27.3%). The biostimulant group with the lowest yield enhancement was Phi (+8.6%; CI 4.6–12.5%), and it was derived from the smallest dataset with 18 comparisons from three studies. The other four biostimulant categories, Chi, HFA, PHs, and SWE, showed an intermediate increase of 14.8–17.1%. Regarding the impact of the commercial status of biostimulants, non-marketed biostimulants tended to represent a stronger yield enhancement effect (+21.8%; CI 20.0–23.5%) than commercially purchased products (+14.4%; CI 12.7–16.0%). For SWE, commercial or non-commercial sources resulted in a similar yield increase of about 16.5–18.0%. The variation in results was the largest for Si, while SWE showed the most consistent yield increase.

### Yield Effectiveness Affected by Biostimulant Application Method

**Figure 3:**
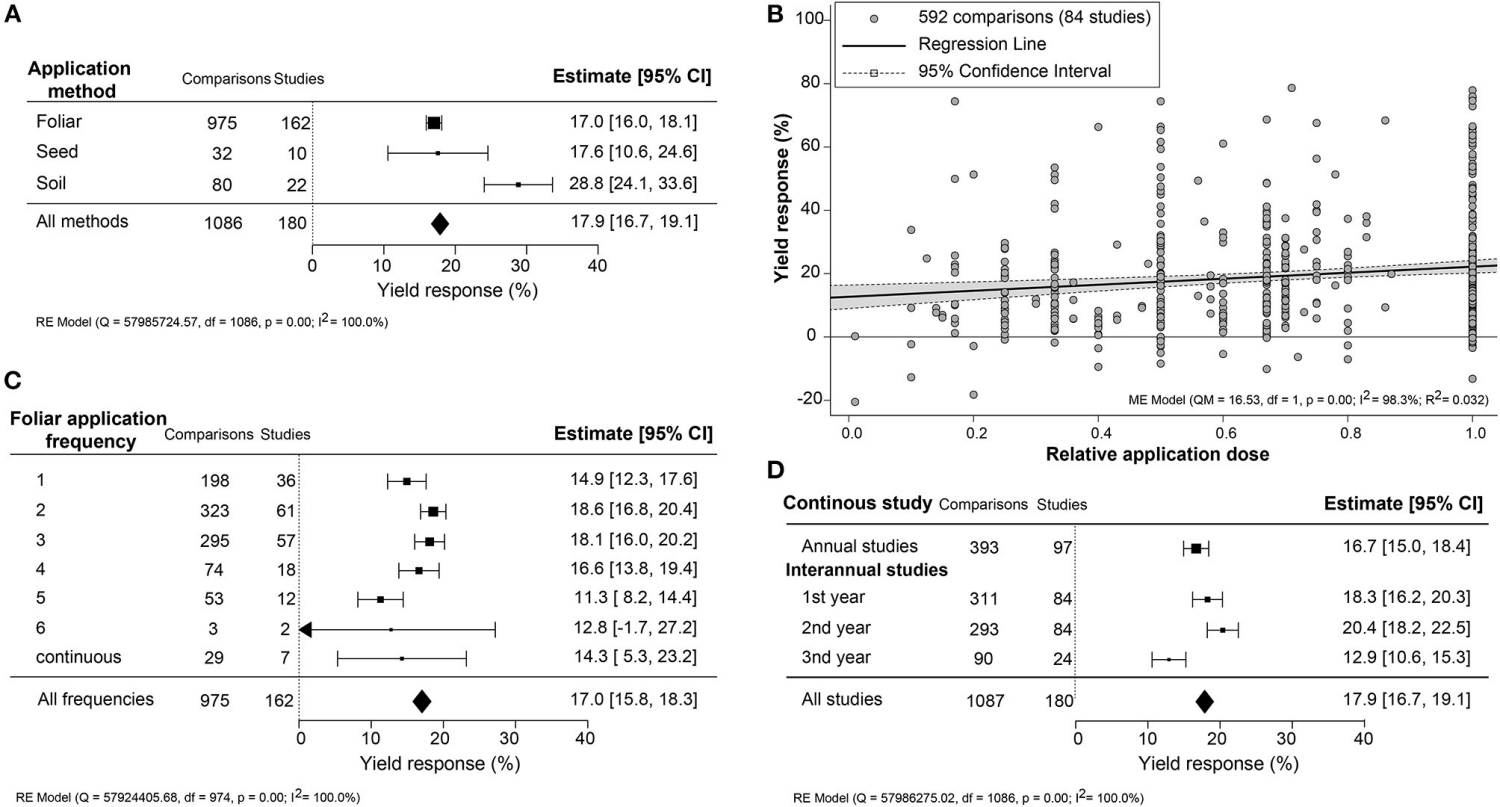
Percentage yield response to biostimulant application affected by the different application management practices, including **(A)** application method, **(B)** frequency, **(C)** concentration, and **(D)** interannual studies. The point size correlates to the estimate's precision, and the error bars represent 95% confidence intervals (CI) of mean estimated effect sizes. The number of comparisons and studies is indicated in each line or legend. The combined effect estimates and the test of heterogeneity on the models [random-effect models (RE) in **(A,B,D)**, and mixed-effect model (ME) in **(C)**] were summarized at the bottom, where the heterogeneity test is significant (*p* < 0.001) and *I*^2^ ≥ 75% implies substantial heterogeneity.

Since the application methodology is an important efficacy determinant, the yield increase across different application methods (foliar, seed, and soil) and associated variables (frequency, dose, and interannual application) were compared (Figure 3). An unexpected result was that soil treatment, an indirect application method, resulted in the most substantial yield increase (+28.8%; CI 24.0–33.6%). Foliar treatments, representing over 85% of the studies, and seed application were similar in an average yield increase of about 17.0% (Figure 3A). In several studies, different dilutions of the biostimulant were tested. Here, we set the highest dose to “1” and calculated the effectiveness of dose responses (Figure 3B). The positive yield effect increased with higher doses, as was expected. However, the regression slope was very shallow, indicating that the doses applied were, in general, close to the saturation level. Single spray applications resulted in a comparably stronger yield increment (+14.9%; CI 12.3–17.6%) with subsequent sprayings of up to four times, resulting in only a slight further increase in yield reaching 16.6–18.6% (Figure 3C). More frequent spraying was counterproductive, with lower yield benefits between 11.3 and 14.3%. When comparing the use of biostimulant within annual or continuous crop production studies, a slightly higher yield improvement in the first 2 years of interannual cultivation was observed (~18.3–20.4%) rather than the single growth season (+16.7%; CI 15.0–18.4%) (Figure 3D). However, the efficiency of biostimulant application decreased in the third year (+12.9%; CI 10.6–15.3%) in interannual studies.

### Vegetables Respond the Most to Yield Improvement

**Figure 4:**
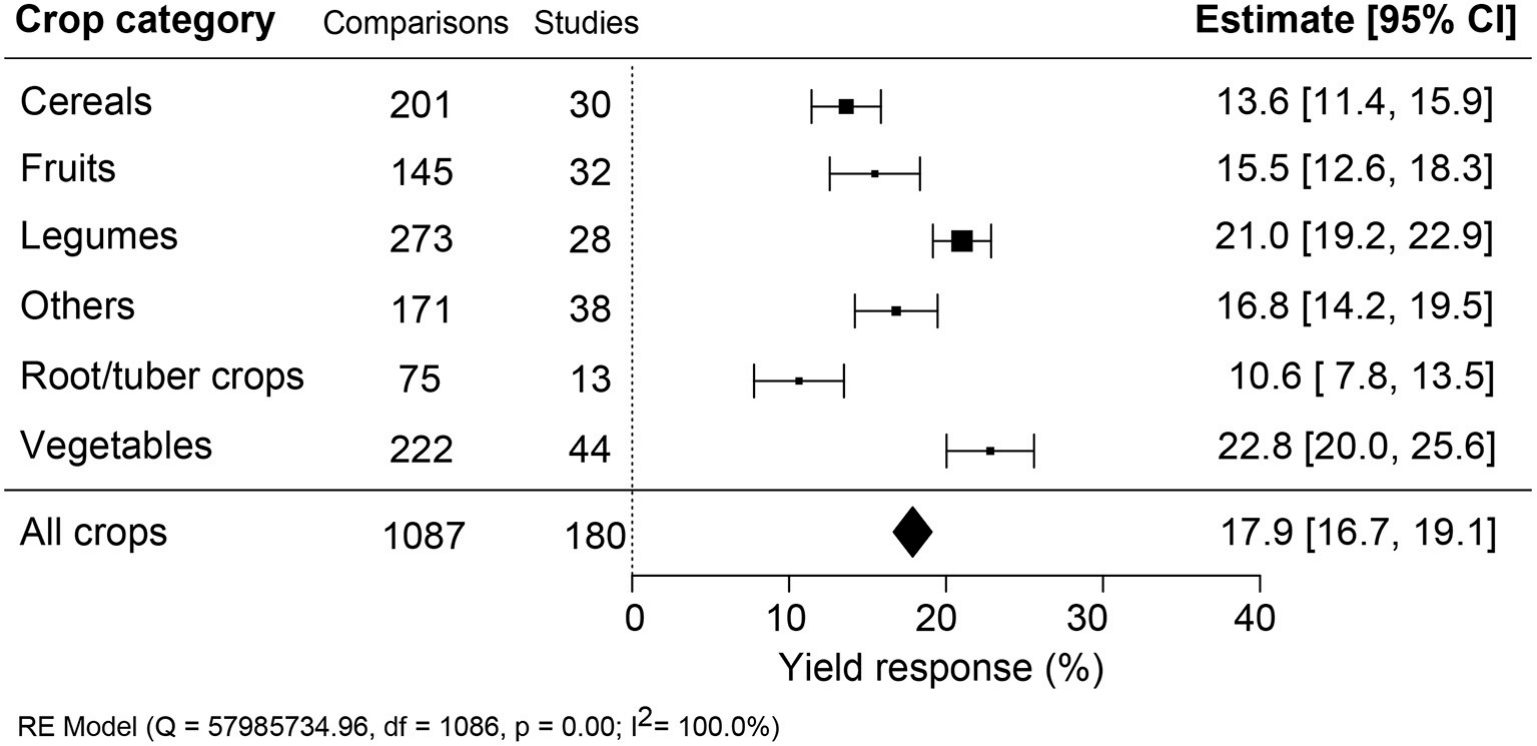
Percentage yield response to biostimulant application affected by the crop categories. The point size correlates to the estimate's precision, and the error bars represent 95% confidence intervals (CI) of mean estimated effect sizes. The number of comparisons and studies is indicated in each line. The combined effect estimates and the heterogeneity test on the random-effect model (RE) were summarized at the bottom, where the heterogeneity test is significant (*p* < 0.001) and *I*^2^ ≥ 75% implies substantial heterogeneity.

The effectiveness of biostimulant application was compared across different crop types: cereals, fruits, legumes, root/tubers, vegetables, and other crops (Figure 4). A sound comparison was possible because the number of studies was similar across the different crop types. Vegetable crops showed the highest and roots/tubers the lowest yield benefit, differing by more than two-fold (+22.8% compared to +10.6%). Legumes were significantly better at responding to biostimulant applications than fruits, cereals, and other crops.

### Yield Effectiveness Varied in Climate and Soil Properties

**Figure 5:**
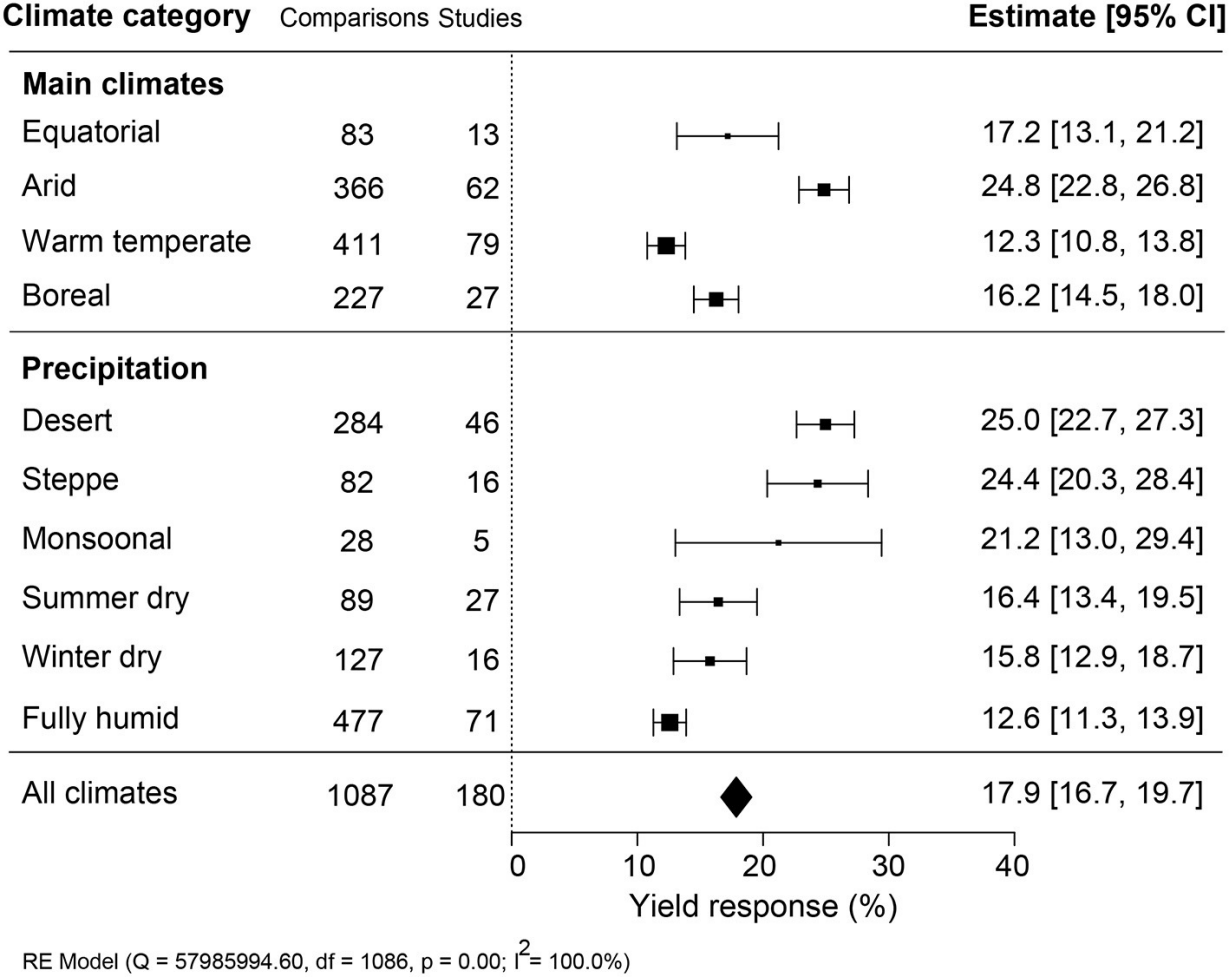
Percentage yield response to biostimulant application affected by the climate categories that were subgrouped into main climates and precipitation types. The point size correlates to the estimate's precision, and the error bars represent 95% confidence intervals (CI) of mean estimated effect sizes. The number of comparisons and studies is indicated in each line. The combined effect estimates and the heterogeneity test on the random-effect model (RE) were summarized at the bottom, where the heterogeneity test is significant (*p* < 0.001) and *I*^2^ ≥ 75% implies substantial heterogeneity.

**Figure 6:**
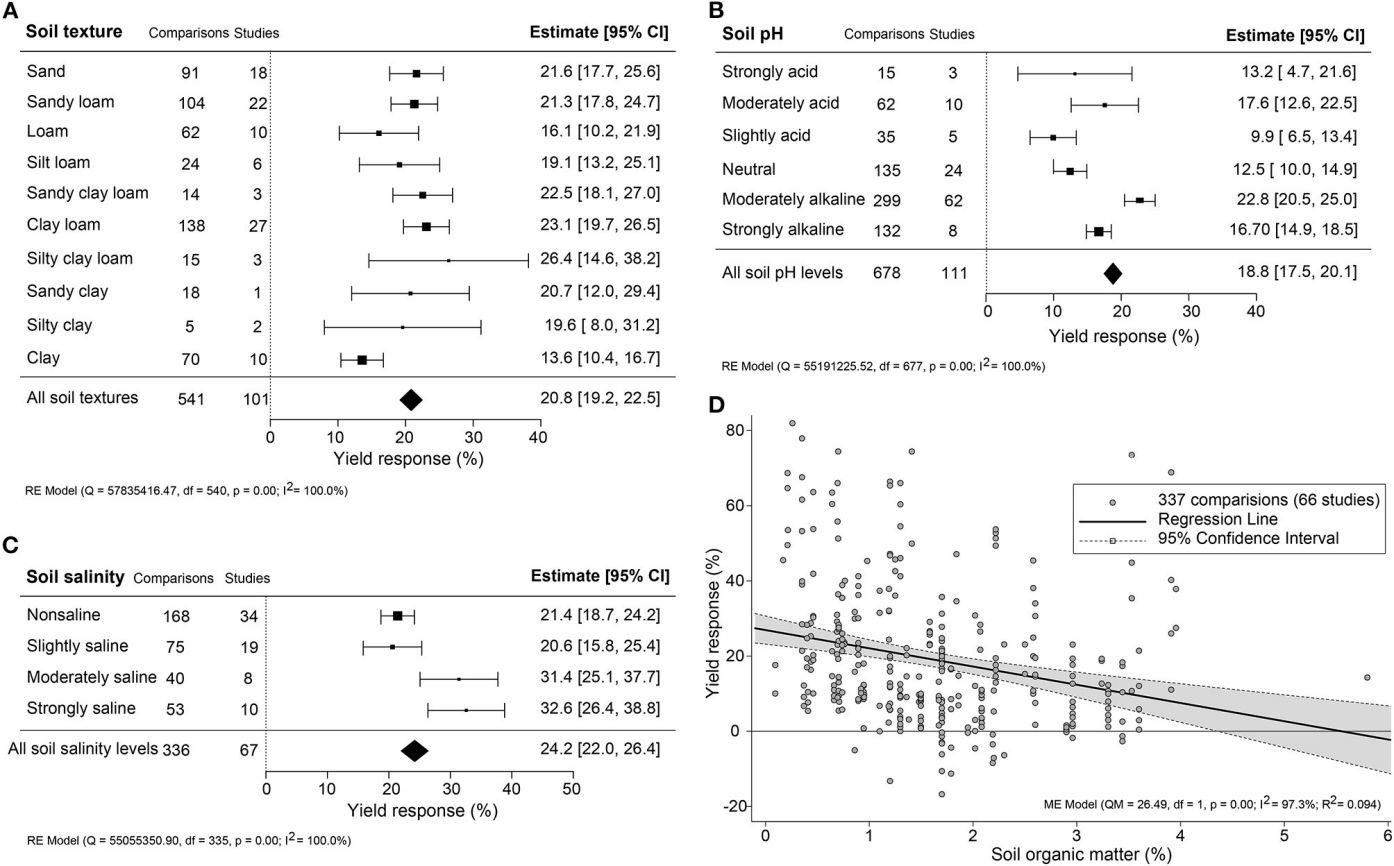
Percentage yield response to biostimulant application affected by the soil properties, including soil **(A)** texture, **(B)** pH, **(C)** salinity, and **(D)** organic matter (%). The point size correlates to the estimate's precision, and the error bars represent 95% confidence intervals (CI) of mean estimated effect sizes. The number of comparisons and studies is indicated in each line or each legend. The combined effect estimates and the heterogeneity test on the models [random-effect models (RE) in **(A**–**C)**, and mixed-effect model (ME) in **(D)**] were summarized at the bottom, where the heterogeneity test is significant (*p* < 0.001) and *I*^2^ ≥ 75% implies substantial heterogeneity.

**Figure 7:**
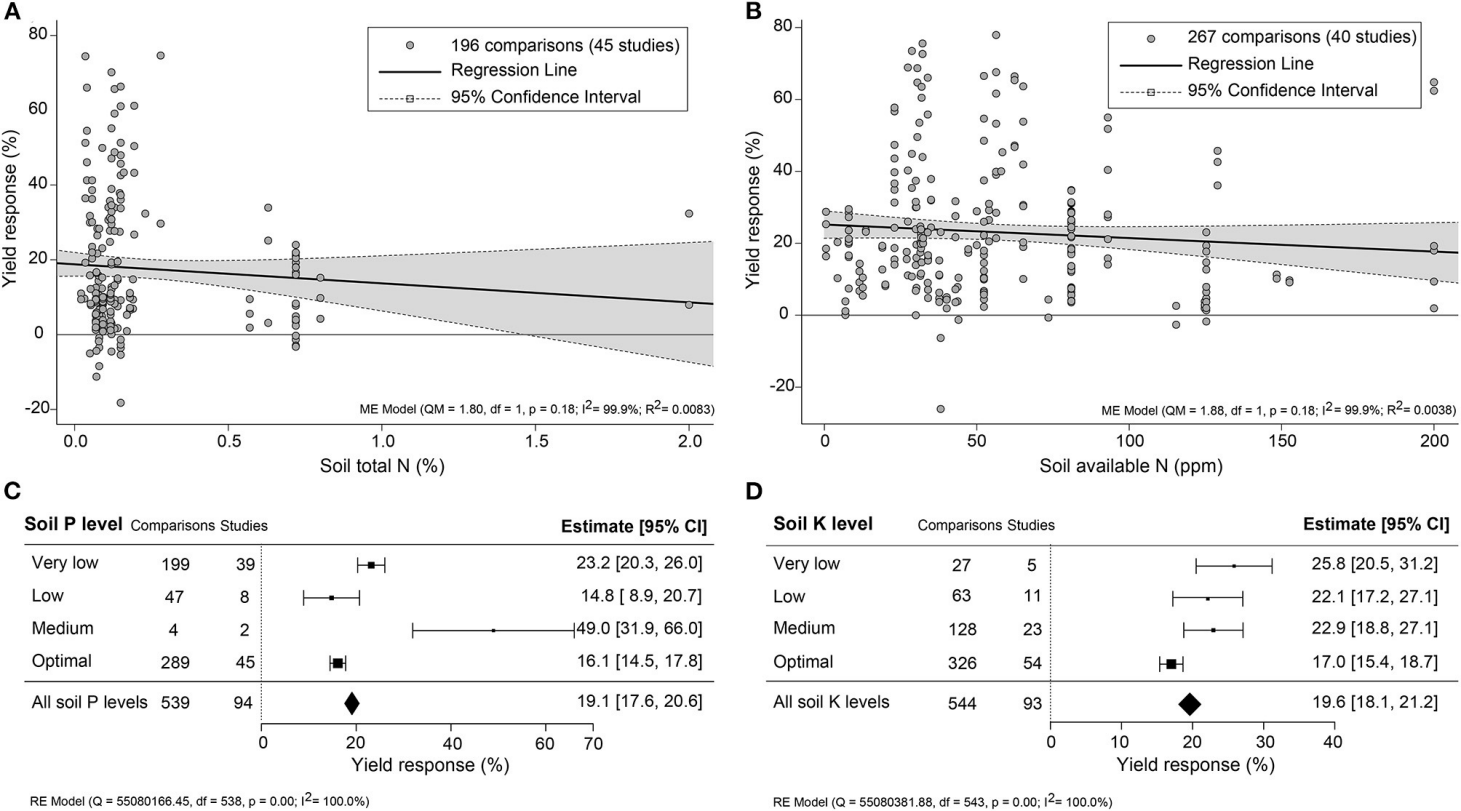
Percentage yield response to biostimulant application as affected by the macronutrient levels, including **(A)** soil total N (%), **(B)** soil available N (ppm), soil **(C)** P, and **(D)** K levels. The point size correlates to the estimate's precision, and the error bars represent 95% confidence intervals (CI) of mean estimated effect sizes. The number of comparisons and studies is indicated in each line or each legend. The combined effect estimates and the heterogeneity test on the models [mixed-effect models (ME) in **(A,B)**, and random-effect models (RE) in **(C,D)**] were summarized at the bottom, where the heterogeneity test is significant (*p* < 0.001) and *I*^2^ ≥ 75% implies substantial heterogeneity.

The impact of climate conditions on biostimulant performance was analyzed by comparing the yield increase across four main climate categories (equatorial, arid, warm temperate, and boreal) and six precipitation types (desert, steppe, monsoonal, summer dry, winter dry, and fully humid) (Figure 5). The effect of biostimulant was most positive in climates with seriously limited water availability (arid and desert). Moreover, yield gain showed a clear negative trend with increased precipitation, with fully humid climate conditions as the least favorable for biostimulant efficiency. Overall, water availability was revealed as a critical factor, positively correlating with the effect of biostimulants. At the same time, temperature negatively impacted the more extreme side of the spectrum. Next, we compared soil physical and chemical parameters: textures, pH, salinity, and SOM (Figure 6). For most soil types, the average effect of biostimulant was within the same interval range. However, pure clay had a clear lower impact (+13.5%), even though soils with a high clay component were among the best scoring soils (e.g., silty clay loam; +26.3%) (Figure 6A). In general, regarding the soil pH levels, mild soil acidity or alkalinity were better than soils with a neutral or more extreme low or high pH (Figure 6B). Moderate alkaline soil reveals the highest potential response to biostimulant application. Soil salinity strongly positively correlated with biostimulant effectiveness (Figure 6C), in line with the water availability correlation shown in Figure 5. Finally, we compared biostimulant effectiveness across soils with different SOM content. Here, we found a robust negative trend between SOM and yield response after biostimulant application (Figure 6D). A negative correlation was also observed with increasing soil total N (%) and soil available N content (ppm) (Figures 7A,B). Concerning soil P and K levels, the analysis revealed that biostimulants function better in poor soils deficient in P and K nutrients (Figures 7C,D).

## Discussion

### Biostimulant Effectiveness

A large body of published data demonstrates the positive impact of numerous types of biostimulants on a wide range of crops using different application methods under various conditions. However, for biostimulants to become standard practice, these products will require consistency and reproducibility in the beneficial effects they are claimed to have on crop production. A standardization for measuring the effectiveness of biostimulants is required to distinguish the good from the bad (Ricci et al., 2019). Variation in biostimulant effectiveness is expected as different crops respond differently to biostimulants, and the environmental conditions are likely also influencing the effects. Therefore, a one-to-one comparison of biostimulant effectiveness based on published data is not likely to be very reliable. In this study, we looked at the bigger picture and queried the literature for data published on the effectiveness of biostimulants. To allow for sound comparisons, the study focused on biostimulants derived from natural resources categorized according to their origin and chemical properties (Geelen and Xu, 2020) and restricted to crop yield experiments in open fields that are closer to an application and commercialization target. The main result from the meta-analysis was that it revealed correlations between biostimulant effectiveness and impactors that have, insofar as we are aware, not previously been completely recognized.

Since most authors wish to report successful biostimulants, it is fair to assume that publications are biased toward the positive. This does, however, not prevent the identification of correlations between the effects and the materials and application conditions used. With the high yield gain found, one would expect widespread use of biostimulants in many crop production systems. This is currently not the case, and we, therefore, assume that the average yield increase reported here is an overestimation of what can be expected in a commercial context. Noteworthy here is that the efficiency of non-commercial products was 7% higher than commercial ones (Figure 2). Indeed, a more conservative estimation of the yield increase is warranted, and there is a need for a more systematic collection of yield data to conclude the effectiveness of commercial crop production systems (Geelen and Xu, 2020). Previously, a meta-analysis focusing on humic substances under controlled environment and field studies reported an estimated just above 20% of the increase in dry weight of shoot and root (Rose et al., 2014), which is close to the yield gain we found (+17.9%) (Figure 2). In the case of microbial PBs, Schütz et al. (2018) found that the yield benefit in field trials was between +8.5 and +20.0%. Taken together, the published data show some level of consistency across the different biostimulants analyzed. Chemical fertilizers also contribute to a yield gain, and here the average contribution was estimated to be around 40–60% (Stewart et al., 2005). Considering that biostimulants are commonly applied as supplements under conventional fertilization schemes and in many cases usually contain NPK fertilizers, the net positive effect of the bioactive ingredients is expected to be a considerable fraction of the total yield gain. Nevertheless, biostimulants sustainably improve the yield and provide a solution to reducing the dependency on synthetic fertilizer.

The extent of yield improvement varied across categories, with PE and MLE as the best performing biostimulants (Figure 2). MLE has been historically tested on many different crops displaying beneficial effects on seed germination, plant growth and yield, nutrient use efficiency, quality traits, and tolerance to abiotic stresses (Zulfiqar et al., 2020). The significant profitability and variability in MLE and other PE efficacy might be due to their complex composition of plant metabolites, containing many macro and mineral nutrients, osmoprotectants, and antioxidants (Soares et al., 2021). PE also likely contains plant hormones, which, in small quantities, are known to harbor the capacity to stimulate crop production (Harms and Oplinger, 1988). None of the MLE products used are, insofar we know, commercialized. This contrasts with SWE, for which more than 60% of the products analyzed are commercialized. Moreover, from over 40% of the total dataset, we estimated the effect of SWE products with more confidence compared to the other biostimulant categories. The consistency in yield benefits linked with SWE application is likely a result of the standardization of SWE extraction and formulation methods. The use of SWE as a plant growth regulator can be traced back as far as the Roman Empire (Henderson, 2004), with the first commercial product marketed in 1952 (Milton, 1952). The technology of SWE production has developed into a mainstream hot-alkaline extraction method involving specific manufacturing conditions, allowing strong consistency in production and quality (Craigie, 2011). However, the more recently discovered biostimulants like PE show batch variations, and their processing methodology is not well-established (García-García et al., 2020).

While many biostimulants are typically complex mixtures, Phi and Si are simple inorganic salts. Despite their less complex chemical composition, the average effectiveness of Phi was the lowest, and that of Si showed the greatest variability. Compared to these products, PBs consisting of complex mixtures were more effective, and it will be a challenge to determine their mechanism of action. It also remains to be demonstrated whether the complex biostimulants exert stronger bioactivity because of synergistic interactions between bioactive ingredients (García-García et al., 2020). Therefore, further standardization in biostimulant production procedures is forecasted to improve the consistency in effectiveness and reproducibility on yield gaining benefits.

### Impact of Biostimulant Application Methodology

Biostimulants applied via soil resulted in about 10% higher yield benefits than foliar and seed applications (Figure 3A). This outcome is surprising as foliar and seed applications deliver the biostimulants directly to the plant, allowing faster uptake of the bioactive ingredients (Niu et al., 2021). For instance, surface spraying acts more directly and results in rapid responses to ripen fruits (Fernández and Eichert, 2009). Soil application of biostimulants likely has a different mode of action related to nutrient uptake efficiency or enhancing microbial activity on and around the crop. Nutrient availability is a major yield factor that can be improved by either providing higher levels of mineral or organic nutrients or by altering the microbial community interacting with the root system (Schütz et al., 2018; Kour et al., 2019; Oldroyd and Leyser, 2020). HFA, PHs, SWE, and PGPR have been shown to stimulate micro and macronutrient uptake efficiency, either by direct activation of ion transporters, mineral utilization, or improving soil quality and mineral recycling (for an extensive review, see Halpern et al., 2015).

Foliar application is the favored method because it can be merged with conventional spraying practices. Remarkably, single biostimulant sprays were nearly as effective as multiple applications (Figure 3C). This suggests that the yield benefit is likely due to nutrient supply and other rapid-growth stimulation induced upon spraying the crop once or twice. Also noteworthy is that applications above 4 times resulted in a negative trend with lower efficiency. The diminishing returns of higher biostimulant application frequencies may be caused by changes in the uptake and assimilation rate of effective agents throughout the germination, vegetative, and reproductive plant developmental stages (Bulgari et al., 2019). Colla et al. (2015) recommended lowering the dosage when frequently applying biostimulant to avoid growth inhibition caused by overdose. In general, the efficiency of biostimulants depends on the plant's nutrient uptake rate, which is highest prior to maximum growth rates depending on the crop type (Jones et al., 2011; Nguyen et al., 2019). For example, SWE application was best during the tilling stage (Stamatiadis et al., 2021) and best during the seedling stage of sugarcane (Chen et al., 2021). Moreover, the sensitivity of plants is regulated by their daily circadian clock that also may influence the effectiveness of the biostimulant (Belbin et al., 2019). It is thus suggested to spray biostimulants in the early morning or late afternoon because of the open stomata (Specialty Fertilizers, 2015). Summarily, we strongly advise following the optimized biostimulant application in crop management.

### Comparison Between Crop Categories

Vegetable and legume crops showed the highest gain in yield upon biostimulant application (Figure 4). A previous meta-analysis study on the crop yield improvement via biofertilization with microbial PBs argued that vegetables require higher fertilizer concentrations for optimal growth, and legumes engage in symbiotic nitrogen fixation, which is stimulated upon the addition of microbial PBs (Schütz et al., 2018). As our analysis included only non-microbial PBs, the stronger legume response is not likely attributed to the stimulation of symbiotic interactions with nitrogen-fixing bacteria. It is currently unclear why vegetable and legume crops are more responsive to biostimulant application.

### Biostimulants Are More Efficient Under Suboptimal Growing Conditions

Overall, biostimulants showed the strongest crop yield effects in soils of low quality (acid and alkaline soils, saline soils, barren soils with low SOM, and P- or K-deficient soils) (Figures 6B–D, 7C,D). In these soils, the cation exchange capacity (CEC) is inherently nutrient-poor (Brown and Lemon, 2021). Soil rich in clay component or SOM, for instance, has a higher CEC value and typically retains higher levels of nutrients, and therefore supporting sustained crop growth and higher yield (Bayu, 2020). The yield gap of cultivation in poor soils is thus much larger than in fertile soils (Evans and Fischer, 1999). Therefore, we suggest combining biostimulant applications with “Integrated Fertility Management” to maximize yield potential and reduce crop loss risk under climate change scenarios.

Open-field production systems are exposed to variations in climate conditions and, therefore, at risk of abiotic and biotic stresses and degradation of the soil conditions (drought, salinity, nutrient deficiency) (Challinor et al., 2014; Mickelbart et al., 2015). Biostimulants are propagated as a solution to safeguard crop yield under suboptimal growth conditions (Yakhin et al., 2017). In agreement with this view, the effectiveness of biostimulant application was the highest under suboptimal growing conditions of arid climates with low precipitation conditions (Figure 5). A similar conclusion was made from a meta-analysis on the yield improvement using microbial PBs (Schütz et al., 2018). In arid climate conditions, crops are exposed to more extreme temperature conditions, which strongly impacts crop fertility (Thakur et al., 2010; Deryng et al., 2014; De Storme and Geelen, 2014). How PBs can mitigate environmental stresses is not well understood. Exogenously applied compounds may elicit a stress response that prepares the plant for subsequent stresses caused by limitations in water, soil fertility, or unfavorable temperature conditions (Ahmad et al., 2019). Molecules that can elicit a stress response are present in biostimulants and have been shown to induce stress-related genes (Geelen and Xu, 2020; González-Morales et al., 2021). Phytohormones are also commonly present in biostimulants, and their interactions with plants are known to enhance osmolyte accumulation and tolerance to stress (Sharma et al., 2019). In addition, plant-derived biostimulants contain antioxidants and improve the adaptation to unfavorable growing conditions by eliminating reactive oxygen species (ROS) (Drobek et al., 2019). Therefore, the bioactive compounds in biostimulants may evoke either stress alleviation (e.g., suppression of ROS) or induce stress response factors that trigger the immunity against abiotic stresses (Brown and Saa, 2015), validating the hypothesis that biostimulants are more effective under suboptimal growth conditions.

## Conclusions

This review underscores the importance of evaluating the biostimulant application methodology and the crop cultivation conditions. The study indicates that the impact of biostimulant application on crop yield depends on the type of products and application management. Our results also provide various environment-specific assessments of biostimulant performance in open-field conditions, which can be used to set up more effective farming practices for future biostimulant application strategies. In conclusion, biostimulants improve crop yield by reducing yield reductions under stress conditions. This approach can help improve food security for the growing world population under increasing climate change threats.

## Data Availability Statement

The original contributions presented in the study are included in the article/Supplementary Material, further inquiries can be directed to the corresponding author/s.

## Author Contributions

JL: conceptualization, investigation, formal analysis, data curation, visualization, and writing—original draft. TV: writing—review and editing and supervision. DG: conceptualization, funding acquisition, writing—review and editing, and supervision. All authors contributed to the article and approved the submitted version.

## Funding

This research was supported by Fonds Wetenschappelijk Onderzoek – Vlaanderen (FWO) under project Bio2Bio (S006017N) and under project BioSUNmulant by European Union's Horizon 2020 research and innovation program under grant agreement of sustainable and resilient agriculture for food and non-food systems (FACCE SURPLUS, No. 652615) and with Flanders Innovation & Entrepreneurship (VLAIO) (HBC.2019.2244). JL was supported by the China Scholarship Council (CSC) Grant No. 201706350259.

## Conflict of Interest

The authors declare that the research was conducted in the absence of any commercial or financial relationships that could be construed as a potential conflict of interest.

## Publisher's Note

All claims expressed in this article are solely those of the authors and do not necessarily represent those of their affiliated organizations, or those of the publisher, the editors and the reviewers. Any product that may be evaluated in this article, or claim that may be made by its manufacturer, is not guaranteed or endorsed by the publisher.
