# Supplementary material for: A Meta-Analysis of Biostimulant Yield Effectiveness in Field Trials
Source: Front Plant Sci. 2022 Apr 14;13:836702. doi: 10.3389/fpls.2022.836702 (PMC9047501; doi:10.3389/fpls.2022.836702)
Supplement: Supplementary file 1 [file Table_2.DOCX]

**Identification of studies via databases and registers**

Records removed *before screening*:

Duplicate records removed (n = 468)

Records identified from*:

Databases (n = 859)

**Identification**

Records screened

(n = 391)

Records excluded**

(n = 0)

Reports sought for retrieval

(n = 391)

Reports not retrieved

(n = 23)

**Screening**

Reports assessed for eligibility

(n = 368)

Reports excluded:

Full-text unavailable (n = 18)

Imcomplete information (n = 4)

Not written in english (n = 2)

Greenhouse study (n = 120)

Non-marketable yield (n = 43)

Studies included in review

(n = 181)

Reports of included studies

(n = 180)

**Included**

*Consider, if feasible to do so, reporting the number of records identified from each database or register searched (rather than the total number across all databases/registers).

**If automation tools were used, indicate how many records were excluded by a human and how many were excluded by automation tools.

*From:*  Page MJ, McKenzie JE, Bossuyt PM, Boutron I, Hoffmann TC, Mulrow CD, et al. The PRISMA 2020 statement: an updated guideline for reporting systematic reviews. BMJ 2021;372:n71. doi: 10.1136/bmj.n71

For more information, visit: <http://www.prisma-statement.org/>
