# Supplementary material for: A Meta-Analysis of Biostimulant Yield Effectiveness in Field Trials
Source: Front Plant Sci. 2022 Apr 14;13:836702. doi: 10.3389/fpls.2022.836702 (PMC9047501; doi:10.3389/fpls.2022.836702)
Supplement: Supplementary file 2 [file Data_Sheet_1.DOCX]

**Supplementary Data 1.** Studies included for meta-analysis.

1. Abbas, M., Anwar, J., Zafar-Ul-Hye, M., Iqbal Khan, R., Saleem, M., Rahi, A.A., Danish, S., and Datta, R. (2020). Effect of Seaweed Extract on Productivity and Quality Attributes of Four Onion Cultivars. Horticulturae 6.
2. Abdel-Mawgoud, A., Tantawy, A., El-Nemr, M., and Sassine, Y. (2010). Growth and yield responses of strawberry plants to chitosan application. European Journal of Scientific Research 39, 170-177.
3. Abood, M.A., Abd Al-Shammari, A.M., and Hamdi, G.J. (2019). FOLIAR APPLICATION OF TECAMIN MAX (R) TO ALLEVIATE WATER DEFICIT EFFECTS ON YIELD AND WATER-USE EFFICIENCY (WUE) OF OKRA. Acta Scientiarum Polonorum-Hortorum Cultus 18, 15-20.
4. Aghaei, K., Pirbalouti, A.G., Mousavi, A., Badi, H.N., and Mehnatkesh, A. (2019). Effects of foliar spraying of L-phenylalanine and application of bio-fertilizers on growth, yield, and essential oil of hyssop Hyssopus officinalis 1. subsp. Angustifolius (Bieb.). Biocatalysis and Agricultural Biotechnology 21.
5. Ahmed, Y., and Shalaby, E. (2012). Effect of different seaweed extracts and compost on vegetative growth, yield and fruit quality of cucumber. J. Hortic. Sci. Orna. Plants 4, 235-240.
6. Alabdulla, S.A. (2019). Effect of foliar application of humic acid on fodder and grain yield of oat (Avena sativa l.). Research on Crops 20, 880-885.
7. Alam, M.Z., Braun, G., Norrie, J., and Mark Hodges, D. (2014). Ascophyllum extract application can promote plant growth and root yield in carrot associated with increased root-zone soil microbial activity. Canadian Journal of Plant Science 94, 337-348.
8. Al-Bayati, A., Jaafar, H., and Alhasnawi, N. (2020). Evaluation of Eggplant via Different Drip Irrigation Intervals and Foliar Sprays with Seaweed Extract Biostimulant. International Journal of Agricultural and Statistical Sciences 16, 633-639.
9. Ali, O., Ramsubhag, A., and Jayaraman, J. (2019). Biostimulatory activities of Ascophyllum nodosum extract in tomato and sweet pepper crops in a tropical environment. PloS one 14, e0216710.
10. Almadi, L., Paoletti, A., Cinosi, N., Daher, E., Rosati, A., Di Vaio, C., and Famiani, F. (2020). A Biostimulant Based on Protein Hydrolysates Promotes the Growth of Young Olive Trees. Agriculture 10, 618.
11. Al-Shammari, A.M.A., Abood, M.A., and Hamdi, G.J. (2019). Foliar application of Tecamin max® to alleviate water deficit on vegetative growth and yield of okra. International Journal of Vegetable Science 25, 278-284.
12. Al-Tawaha, A., Seguin, P., Smith, D., and Beaulieu, C. (2006). Foliar application of elicitors alters isoflavone concentrations and other seed characteristics of field-grown soybean. Canadian journal of plant science 86, 677-684.
13. Amer, H.M., Marrez, D.A., Salama, A.B., Wahba, H.E., and Khalid, K.A. (2019). Growth and chemical constituents of cardoon plant in response to foliar application of various algal extracts. Biocatalysis and Agricultural Biotechnology 21.
14. Ananthi, K., and Vanangamudi, M. (2013). FOLIAR APPLICATION OF HUMIC ACID WITH BRASSINOSTEROID ON CHLOROPHYLL CONTENT AND YIELD OF GREENGRAM VIGNA RADIATA (L.) WILCZEK. Legume Research 36, 241-244.
15. Arif, M., Kareem, S.H.S., Ahmad, N.S., Hussain, N., Yasmeen, A., Anwar, A., Naz, S., Iqbal, J., Shah, G.A., and Ansar, M. (2019). Exogenously Applied Bio-Stimulant and Synthetic Fertilizers to Improve the Growth, Yield and Fiber Quality of Cotton. Sustainability 11.
16. Atta, M.M.M., Abdel-Lattif, H.M., and Absy, R. (2017). Influence of biostimulants supplement on maize yield and agronomic traits. Bioscience Research 14, 604-615.
17. Atteya, A.K.G., and El Gendy, A.G. (2018). Impact of actosol and yeast extract on productivity and essential oil constituents of Zinnia elegans plants. Bioscience Research 15, 1542-1558.
18. Azarpour, E., Motamed, M.K., Moraditochaee, M., and Bozorgi, H.R. (2012). Effects of bio, mineral nitrogen fertilizer management, under humic acid foliar spraying on fruit yield and several traits of eggplant (Solanum melongena L.). African Journal of Agricultural Research 7, 1104-1109.
19. Bakry, A.B., Taha, M.H., El-Karamany, M.F., and Said, M.T. (2016). Improving Productivity and Quality of Two Wheat Cultivars Using Humic Acid and Zinc Foliar Application under Sandy Soil Conditions. Research Journal of Pharmaceutical Biological and Chemical Sciences 7, 606-618.
20. Baqir, H., and Al-Naqeeb, M.a.S. (2018). RESPONSE OF GROWTH AND YIELD OF THE THREE BREAD WHEAT CULTIVARS TO APPLYING YEAST POWDER METHODS IN DIFFERENT CONCENTRATIONS. International Journal of Agricultural and Statistical Sciences 14, 327-336.
21. Bavaresco, L., Lucini, L., Squeri, C., Zamboni, M., and Frioni, T. (2020). Protein hydrolysates modulate leaf proteome and metabolome in water-stressed grapevines. Scientia Horticulturae 270, 109413.
22. Befrozfar, M.R., Habibi, D., Asgharzadeh, A., Sadeghi-Shoae, M., and Tookalloo, M.R. (2013). Vermicompost, plant growth promoting bacteria and humic acid can affect the growth and essence of basil (Ocimum basilicum L.). Ann Biol Res 4, 8-12.
23. Boselli, M., Bahouaoui, M.A., Lachhab, N., Sanzani, S.M., Ferrara, G., and Ippolito, A. (2019). Protein hydrolysates effects on grapevine (Vitis vinifera L., cv. Corvina) performance and water stress tolerance. Scientia Horticulturae 258.
24. Bozorgi, H.R. (2012). Effects of foliar spraying with marine plant Ascophyllum nodosum extract and nano iron chelate fertilizer on fruit yield and several attributes of eggplant (Solanum melongena L.). ARPN Journal of Agricultural and Biological Science 7, 357-362.
25. Caruso, G., De Pascale, S., Cozzolino, E., Cuciniello, A., Cenvinzo, V., Bonini, P., Colla, G., and Rouphael, Y. (2019). Yield and nutritional quality of Vesuvian piennolo tomato PDO as affected by farming system and biostimulant application. Agronomy 9.
26. Castellano-Hinojosa, A., Meyering, B., Nuzzo, A., Strauss, S.L., and Albrecht, U. (2021). Effect of plant biostimulants on root and plant health and the rhizosphere microbiome of citrus trees in huanglongbing-endemic conditions. Trees.
27. Chen, D., Zhou, W., Yang, J., Ao, J., Huang, Y., Shen, D., Jiang, Y., Huang, Z., and Shen, H. (2021). Effects of Seaweed Extracts on the Growth, Physiological Activity, Cane Yield and Sucrose Content of Sugarcane in China. Frontiers in Plant Science 12.
28. Chouliaras, V., Tasioula, M., Chatzissavvidis, C., Therios, I., and Tsabolatidou, E. (2009). The effects of a seaweed extract in addition to nitrogen and boron fertilization on productivity, fruit maturation, leaf nutritional status and oil quality of the olive (Olea europaea L.) cultivar Koroneiki. Journal of the Science of Food and Agriculture 89, 984-988.
29. Ciepiela, G.A., and Godlewska, A. (2019). The effect of biostimulants derived from various materials on the yield and selected organic components of italian rye grass (lolium multiflorum lam.) against the background of nitrogen regime. Applied Ecology and Environmental Research 17, 12407-12418.
30. Ciepiela, G.A., Godlewska, A., and Jankowska, J. (2013). THE EFFECT OF BIOSTIMULANT ON YIELDS OF MIXED GRASS/RED CLOVER STANDS AND CHLOROPHYLL CONTENT IN CROP PLANT LEAVES UNDER DIFFERENT NITROGEN FERTILISATION REGIMES. Fresenius Environmental Bulletin 22, 3700-3708.
31. Colavita, G.M., Spera, N., Blackhall, V., and Sepulveda, G.M. (2011). Effect of Seaweed Extract on Pear Fruit Quality and Yield. Acta Horticulturae, 601-607.
32. Consentino, B.B., Virga, G., La Placa, G.G., Sabatino, L., Rouphael, Y., Ntatsi, G., Iapichino, G., La Bella, S., Mauro, R.P., D’anna, F., Tuttolomondo, T., and De Pasquale, C. (2020). Celery (Apium graveolens L.) performances as subjected to different sources of protein hydrolysates. Plants 9, 1-13.
33. Correia, S., Queirós, F., Ferreira, H., Morais, M.C., Afonso, S., Silva, A.P., and Gonçalves, B. (2020). Foliar Application of Calcium and Growth Regulators Modulate Sweet Cherry (Prunus avium L.) Tree Performance. Plants 9, 410.
34. Culver, M. (2012). Effect of Moringa oleifera leaf aqueous extract on growth and yield of rape and cabbage. African Journal of Biotechnology 11.
35. De Carvalho, R.P., Pasqual, M., Silveira, H.R.D., De Melo, P.C., Bispo, D.F.A., Laredo, R.R., and Lima, L.D.S. (2019). "Niagara Rosada" table grape cultivated with seaweed extracts: physiological, nutritional, and yielding behavior. Journal of Applied Phycology 31, 2053-2064.
36. Desoky, E.M., Elrys, A.S., and Rady, M.M. (2019). Integrative moringa and licorice extracts application improves Capsicum annuum fruit yield and declines its contaminant contents on a heavy metals-contaminated saline soil. Ecotoxicology and Environmental Safety 169, 50-60.
37. Di Mola, I., Cozzolino, E., Ottaiano, L., Giordano, M., Rouphael, Y., Colla, G., and Mori, M. (2019). Effect of Vegetal- and Seaweed Extract-Based Biostimulants on Agronomical and Leaf Quality Traits of Plastic Tunnel-Grown Baby Lettuce under Four Regimes of Nitrogen Fertilization. Agronomy 9, 571.
38. Di Mola, I., Cozzolino, E., Ottaiano, L., Nocerino, S., Rouphael, Y., Colla, G., El-Nakhel, C., and Mori, M. (2020). Nitrogen Use and Uptake Efficiency and Crop Performance of Baby Spinach (Spinacia oleracea L.) and Lamb’s Lettuce (Valerianella locusta L.) Grown under Variable Sub-Optimal N Regimes Combined with Plant-Based Biostimulant Application. Agronomy 10, 278.
39. Di Mola, I., Ottaiano, L., Cozzolino, E., Senatore, M., Giordano, M., El-Nakhel, C., Sacco, A., Rouphael, Y., Colla, G., and Mori, M. (2019). Plant-Based Biostimulants Influence the Agronomical, Physiological, and Qualitative Responses of Baby Rocket Leaves under Diverse Nitrogen Conditions. Plants-Basel 8.
40. Di Stasio, E., Cirillo, V., Raimondi, G., Giordano, M., Esposito, M., and Maggio, A. (2020). Osmo-priming with seaweed extracts enhances yield of salt-stressed tomato plants. Agronomy 10.
41. Di-Vaio, C., Cirillo, A., Cice, D., El-Nakhel, C., and Rouphael, Y. (2021). Biostimulant Application Improves Yield Parameters and Accentuates Fruit Color of Annurca Apples. Agronomy 11, 715.
42. Dogra, B., and Mandradia, R.K. (2012). Effect of seaweed extract on growth and yield of onion. International Journal of Farm Sciences 2, 59-64.
43. Dudaš, S., Šola, I., Sladonja, B., Erhatić, R., Ban, D., and Poljuha, D. (2016). The Effect of Biostimulant and Fertilizer on “Low Input” Lettuce Production. Acta Botanica Croatica 75, 253-259.
44. Elankavi, S., Sudhakar, P., Ramesh, S., Baradhan, G., and Jawahar, S. (2019). Methods of sowing and foliar nutrition on yield enhancement in blackgram. Plant Archives 19, 2049-2052.
45. El-Hefny, E.M. (2010). Effect of saline irrigation water and humic acid application on growth and productivity of two cultivars of cowpea (Vigna unguiculata L. Walp). Australian Journal of Basic and Applied Sciences 4, 6154-6168.
46. El-Hoseiny, H.M., Helaly, M.N., Elsheery, N.I., and Alam-Eldein, S.M. (2020). Humic Acid and Boron to Minimize the Incidence of Alternate Bearing and Improve the Productivity and Fruit Quality of Mango Trees. HortScience 55, 1026.
47. Elrys, A.S., and Merwad, A. (2017). Effect of Alternative Spraying with Silicate and Licorice Root Extract on Yield and Nutrients Uptake by Pea Plants. Egyptian Journal of Agronomy 39, 279-292.
48. El-Serafy, R.S., and El-Sheshtawy, A.A. (2020). Effect of nitrogen fixing bacteria and moringa leaf extract on fruit yield, estragole content and total phenols of organic fennel. Scientia Horticulturae 265.
49. El-Sharony, T.F., El-Gioushy, S., and Oa, A. (2015). Effect of Foliar Application with Algae and Plant Extracts on Growth, Yield and Fruit Quality of Fruitful Mango Trees Cv. Fagri Kalan. Journal of horticulture 2, 162.
50. Elzaawely, A.A., Ahmed, M.E., Maswada, H.F., Al-Araby, A.A., and Xuan, T.D. (2018). Growth traits, physiological parameters and hormonal status of snap bean (Phaseolus vulgaris L.) sprayed with garlic cloves extract. Archives of Agronomy and Soil Science 64, 1068-1082.
51. Elzaawely, A.A., Ahmed, M.E., Maswada, H.F., and Xuan, T.D. (2017). Enhancing growth, yield, biochemical, and hormonal contents of snap bean (Phaseolus vulgaris L.) sprayed with moringa leaf extract. Archives of Agronomy and Soil Science 63, 687-699.
52. Enan, S., El-Saady, A.M., and El-Sayed, A.B. (2016). Impact of Foliar Feeding With Alga Extract and Boron on Yield and Quality of Sugar Beet Grown in Sandy Soil. Egyptian Journal of Agronomy 38, 319-336.
53. Eris, A., Sivritepe, H.Ö., and Sivritepe, N. (1995). The Effects of Seaweed Extract (Ascophyllum nodosum) on Yield and Quality Criteria in Peppers. Acta Horticulturae 412, 185-192.
54. Falcón-Rodríguez, A.B., Costales, D., Gónzalez-Peña, D., Morales, D., Mederos, Y., Jerez, E., and Cabrera, J.C. (2017). Chitosans of different molecular weight enhance potato (Solanum tuberosum L.) yield in a field trial. Spanish Journal of Agricultural Research 15.
55. Farouk, S., and El-Metwally, I.M. (2019). Synergistic responses of drip-irrigated wheat crop to chitosan and/or silicon under different irrigation regimes. Agricultural Water Management 226.
56. Farouk, S., Youssef, S.A., and Ali, A.A. (2012). Exploitation of biostimulatants and vitamins as an alternative strategy to control early blight of tomato plants. Asian Journal of Plant Sciences 11, 36-43.
57. Fawzy, Z., El-Shal, Z., Li, Y., Zhu, O., and Sawan, O.M. (2012). Response of garlic (Allium sativum, L.) plants to foliar spraying of some bio-stimulants under sandy soil condition. Journal of applied sciences research, 770-776.
58. Fichhof, W.H., Silva, R.D.A., De Oliveira, L.S., and Da Silva, R.M. (2018). Management of Biostimulant and Silicon in Mineral Nutrition and Quality of Cotton Fiber. Journal of Agricultural Science 10.
59. Frioni, T., Tombesi, S., Quaglia, M., Calderini, O., Moretti, C., Poni, S., Gatti, M., Moncalvo, A., Sabbatini, P., Berrìos, J.G., and Palliotti, A. (2019). Metabolic and transcriptional changes associated with the use of Ascophyllum nodosum extracts as tools to improve the quality of wine grapes (Vitis vinifera cv. Sangiovese) and their tolerance to biotic stress. Journal of the Science of Food and Agriculture 99, 6350-6363.
60. Gajc-Wolska, J., Kowalczyk, K., Nowecka, M., Mazur, K., and Metera, A. (2012). Effect of organic-mineral fertilizers on the yield and quality of endive (Cichorium endivia L.). Acta Scientiarum Polonorum, Hortorum Cultus 11.
61. Garmendia, A., Raigon, M.D., Marques, O., Ferriol, M., Royo, J., and Merle, H. (2018). Effects of nettle slurry (Urtica dioica L.) used as foliar fertilizer on potato (Solanum tuberosum L.) yield and plant growth. Peerj 6.
62. Głosek-Sobieraj, M., Cwalina-Ambroziak, B., and Hamouz, K. (2018). The Effect of Growth Regulators and a Biostimulator on the Health Status, Yield and Yield Components of Potatoes (Solanum tuberosum L.). Gesunde Pflanzen 70, 1-11.
63. Godlewska, A., and Ciepiela, G. (2020). Yield Performance and Content of Selected Organic Compounds in Trifolium pratense Treated with Various Biostimulants against the Background of Nitrogen Fertilisation. Legume Research: An International Journal 43.
64. Godlewska, A., and Ciepiela, G.A. (2016). The effect of growth regulator on dry matter yield and some chemical components in selected grass species and cultivars. Soil Science and Plant Nutrition 62, 297-302.
65. Godlewska, A., and Ciepiela, G.A. (2018). Assessment of the effect of various biostimulants on medicago X varia T. Martyn yielding and content of selected organic components. Applied Ecology and Environmental Research 16, 5571-5581.
66. Godlewska, K., Pacyga, P., Michalak, I., Biesiada, A., Szumny, A., Pachura, N., and Piszcz, U. (2020). Field-Scale Evaluation of Botanical Extracts Effect on the Yield, Chemical Composition and Antioxidant Activity of Celeriac (Apium graveolens L. Var. rapaceum). Molecules 25, 4212.
67. Grabowska, A., Kunicki, E., Sękara, A., Kalisz, A., and Wojciechowska, R. (2012). The Effect of Cultivar and Biostimulant Treatment on the Carrot Yield and its Quality. Vegetable Crops Research Bulletin 77, 37-48.
68. Gugala, M., Zarzecka, K., Dolega, H., and Sikorska, A. (2018). Weed Infestation and Yielding of Potato Under Conditions of Varied Use of Herbicides and Bio-Stimulants. Journal of Ecological Engineering 19, 191-196.
69. Hadi, M., Darz, M.T., Ghandehari, Z., and Riazi, G. (2011). Effects of vermicompost and amino acids on the flower yield and essential oil production from Matricaria chamomile L. Journal of Medicinal Plants Research 5, 5611-5617.
70. Haider, M.W., Ayyub, C.M., Pervez, M.A., Asad, H.U., Manan, A., Raza, S.A., and Ashraf, I. (2012). Impact of foliar application of seaweed extract on growth, yield and quality of potato (Solanum tuberosum L.). Soil & Environment 31.
71. Hamada, A., and Hamd-Alla, W.A. (2019). Productivity of Intercropped Wheat with Faba Bean under Crop Sequences and Foliar Application of Humic Acid. Egyptian Journal of Agronomy 41, 225-241.
72. Hameed, A., Fatma, S., Wattoo, J.I., Yaseen, M., and Ahmad, S. (2018). Accumulative effects of humic acid and multinutrient foliar fertilizers on the vegetative and reproductive attributes of citrus (Citrus reticulata cv. kinnow mandarin). Journal of Plant Nutrition 41, 2495-2506.
73. Hammad, S.a.R., and Ali, O.a.M. (2014). Physiological and biochemical studies on drought tolerance of wheat plants by application of amino acids and yeast extract. Annals of Agricultural Sciences 59, 133-145.
74. Hamze, M.R., Khoshgoftarmanesh, A.H., Shariatmadari, H., and Baninasab, B. (2018). The effects of foliar applied potassium in the mineral form and complexed with amino acids on pistachio nut yield and quality. Archives of Agronomy and Soil Science 64, 1432-1445.
75. Han, X., Xi, Y., Zhang, Z., Mohammadi, M.A., Joshi, J., Borza, T., and Wang-Pruski, G. (2021). Effects of phosphite as a plant biostimulant on metabolism and stress response for better plant performance in Solanum tuberosum. Ecotoxicology and Environmental Safety 210.
76. Hidangmayum, A., and Sharma, R. (2017). Effect of different concentrations of commercial seaweed liquid extract of Ascophyllum nodosum as a plant bio stimulant on growth, yield and biochemical constituents of onion (Allium cepa L.). J. Pharmacogn. Phytochem 6, 658-663.
77. Hussein, N., Hussein, M., Gadel Hak, S., Hammad, M., and Shaalan, H. (2014). Effect of two plant extracts and four aromatic oils on tuta absoluta population and productivity of tomato cultivar gold stone. Nat. Sci 12, 108-118.
78. Jadhao, G.R., Chaudhary, D.R., Khadse, V.A., and Zodape, S.T. (2015). Utilization of seaweeds in enhancing productivity and quality of black gram Vigna mungo (L.) Hepper for sustainable agriculture. Indian Journal of Natural Products and Resources 6, 16-22.
79. Kahraman, A. (2017). Effect of humic acid doses on yield and quality parameters of cowpea Vigna unguiculata (L.) Wall) cultivars. Legume Research 40, 155-159.
80. Kandil, A.A., Sharief, A.E., and Fathalla, F.H. (2013). Onion yield as affected by foliar application with amino and humic acids under nitrogen fertilizer levels. Crop Production 2, 62-72.
81. Karimzadeh Asl, K., and Hatami, M. (2019). Application of zeolite and bacterial fertilizers modulates physiological performance and essential oil production in dragonhead under different irrigation regimes. Acta Physiologiae Plantarum 41.
82. Khan, S., Basra, S.M.A., Afzal, I., Nawaz, M., and Rehman, H.U. (2017). Growth promoting potential of fresh and stored Moringa oleifera leaf extracts in improving seedling vigor, growth and productivity of wheat crop. Environmental Science and Pollution Research 24, 27601-27612.
83. Khan, S., Basra, S.M.A., Nawaz, M., Hussain, I., and Foidl, N. (2019). Combined application of moringa leaf extract and chemical growth-promoters enhances the plant growth and productivity of wheat crop (Triticum aestivum L.). South African Journal of Botany.
84. Khazaie, H.R., Rezaie, E.E., and Bannayan, M. (2011). Application times and concentration of humic acid impact on aboveground biomass and oil production of hyssop (Hyssopus officinalis). Journal of Medicinal Plants Research 5, 5148-5154.
85. Kiczorowski, P. (2019). Influence of npk minerals and biostimulants on the growth, yield, and fruit nutritional value in cv. ‘Šampion’ apple trees growing on different rootstocks. Acta Scientiarum Polonorum, Hortorum Cultus 18, 197-205.
86. Knapowski, T., Barczak, B., Kozera, W., Wszelaczynska, E., and Poberezny, J. (2019). Crop stimulants as a factor determining the yield and quality of winter wheat grown in Notec Valley, Poland. Current Science 116, 1009-1015.
87. Kocira, A., Lamorska, J., Kornas, R., Nowosad, N., Tomaszewska, M., Leszczyńska, D., Kozłowicz, K., and Tabor, S. (2020). Changes in Biochemistry and Yield in Response to Biostimulants Applied in Bean (Phaseolus vulgaris L.). Agronomy 10, 189.
88. Kocira, S. (2019). Effect of amino acid biostimulant on the yield and nutraceutical potential of soybean. Chilean Journal of Agricultural Research 79, 17-25.
89. Kocira, S., Szparaga, A., Findura, P., and Treder, K. (2020). Modification of Yield and Fiber Fractions Biosynthesis in Phaseolus vulgaris by Treatment with Biostimulants Containing Amino Acids and Seaweed Extract. Agronomy 10, 1338.
90. Kocira, S., Szparaga, A., Hara, P., Treder, K., Findura, P., Bartoš, P., and Filip, M. (2020). Biochemical and economical effect of application biostimulants containing seaweed extracts and amino acids as an element of agroecological management of bean cultivation. Scientific Reports 10, 17759.
91. Kocira, S., Szparaga, A., Kocira, A., Czerwińska, E., Wójtowicz, A., Bronowicka-Mielniczuk, U., Koszel, M., and Findura, P. (2018). Modeling biometric traits, yield and nutritional and antioxidant properties of seeds of three soybean cultivars through the application of biostimulant containing seaweed and amino acids. Frontiers in Plant Science 9.
92. Kocira, S., Szparaga, A., Kubon, M., Czerwinska, E., and Piskier, T. (2019). Morphological and Biochemical Responses of Glycine max (L.) Merr. to the Use of Seaweed Extract. Agronomy-Basel 9.
93. Kostadinova, S., Kalinova, S., Hristoskov, A., and Samodova, A. (2015). Efficiency of some foliar fertilizers in winter wheat. Bulgarian Journal of Agricultural Science 21, 742-746.
94. Kowalska, J., Tyburski, J., Jakubowska, M., and Krzymińska, J. (2021). Effect of Different Forms of Silicon on Growth of Spring Wheat Cultivated in Organic Farming System. Silicon 13, 211-217.
95. Kuisma, P. (1989). The effect of foliar application of seaweed extract on potato. Agricultural and Food Science 61, 371-377.
96. Kunicki, E., Grabowska, A., Sękara, A., and Wojciechowska, R. (2010). The effect of cultivar type, time of cultivation, and biostimulant treatment on the yield of spinach (Spinacia oleracea L.). Folia Horticulturae 22, 9-13.
97. Layek, J., Das, A., Ramkrushna, G.I., Trivedi, K., Yesuraj, D., Chandramohan, M., Kubavat, D., Agarwal, P.K., and Ghosh, A. (2015). Seaweed sap: a sustainable way to improve productivity of maize in North-East India. International Journal of Environmental Studies 72, 305-315.
98. Lenssen, A.W., Olk, D.C., and Dinnes, D.L. (2019). Application of a Formulated Humic Product Can Increase Soybean Yield. Crop Forage & Turfgrass Management 5.
99. Lobato, M.C., Machinandiarena, M.F., Tambascio, C., Dosio, G.a.A., Caldiz, D.O., Daleo, G.R., Andreu, A.B., and Olivieri, F.P. (2011). Effect of foliar applications of phosphite on post-harvest potato tubers. European Journal of Plant Pathology 130, 155-163.
100. Lola-Luz, T., Hennequart, F., and Gaffney, M. (2014). Effect on yield, total phenolic, total flavonoid and total isothiocyanate content of two broccoli cultivars (Brassica oleraceae var italica) following the application of a commercial brown seaweed extract (Ascophyllum nodosum). Agricultural and Food Science 23, 28-37.
101. Lopez-Mosquera, M., and Pazos, P. (1997). Effects of seaweed on potato yields and soil chemistry. Biological Agriculture & Horticulture 14, 199-205.
102. Machiani, M.A., Rezaei-Chiyaneh, E., Javanmard, A., Maggi, F., and Morshedloo, M.R. (2019). Evaluation of common bean (Phaseolus vulgaris L.) seed yield and quali-quantitative production of the essential oils from fennel (Foeniculum vulgare Mill.) and dragonhead (Dracocephalum moldavica L.) in intercropping system under humic acid application. Journal of Cleaner Production 235, 112-122.
103. Mahmood, N. (2017). Effect of Biostimulants on Growth, Yield and Quality of Bell Pepper Cv. Yolo Wonder. Pakistan Journal of Agricultural Sciences 54, 311-317.
104. Maswada, H.F., Abd El-Razek, U.A., El-Sheshtawy, A.N.A., and Elzaawely, A.A. (2018). Morpho-physiological and yield responses to exogenous moringa leaf extract and salicylic acid in maize (Zea mays L.) under water stress. Archives of Agronomy and Soil Science 64, 994-1010.
105. Mattner, S.W., Milinkovic, M., and Arioli, T. (2018). Increased growth response of strawberry roots to a commercial extract from Durvillaea potatorum and Ascophyllum nodosum. Journal of Applied Phycology 30, 2943-2951.
106. Matysiak, K., Miziniak, W., Kaczmarek, S., and Kierzek, R. (2018). Herbicides with natural and synthetic biostimulants in spring wheat. Ciencia Rural 48.
107. Mehrafarin, A., Qavami, N., Tahmasebi, Z., Naghdi Badi, H., Abdossi, V., and Seif Sahandi, M. (2015). Phytochemical and morpho-physiological responses of lemon balm (Melissa officinalis L.) to biostimulants Application. Journal of Medicinal Plants 3, 29-42.
108. Merwad, A., and Abdel-Fattah, M.K. (2017). Improving productivity and nutrients uptake of wheat plants using Moringa oleifera leaf extract in sandy soil. Journal of Plant Nutrition 40, 1397-1403.
109. Merwad, A.-R.M. (2018). Using Moringa oleifera extract as biostimulant enhancing the growth, yield and nutrients accumulation of pea plants. Journal of Plant Nutrition 41, 425-431.
110. Michalak, I., Chojnacka, K., Dmytryk, A., Wilk, R., Gramza, M., and Rój, E. (2016). Evaluation of supercritical extracts of algae as biostimulants of plant growth in field trials. Frontiers in Plant Science 7.
111. Mondal, M., Puteh, A., Dafader, N., Rafii, M., and Malek, M. (2013). Foliar application of chitosan improves growth and yield in maize. J. Food Agric. Environ 11, 520-523.
112. Mondal, M.M.A., Puteh, A.B., and Dafader, N.C. (2016). Foliar application of chitosan improved morpho-physiological attributes and yield in summer tomato (Solanum lycopersicum). Pakistan Journal of Agricultural Sciences 53, 339-344.
113. Morales-Payan, J.P., and Stall, W. (2003). Papaya (Carica papaya) response to foliar treatments with organic complexes of peptides and amino acids. Proceedings of the Florida State Horticultural Society 116, 30-32.
114. Nasir, M., Khan, A.S., Basra, S.A., and Malik, A.U. (2016). Foliar application of moringa leaf extract, potassium and zinc influence yield and fruit quality of ‘Kinnow’mandarin. Scientia horticulturae 210, 227-235.
115. Norrie, J., Branson, T., and Keathley, P.E. (2002). "Marine plant extracts impact on grape yield and quality", in: Acta Horticulturae.).
116. Nurdiawati, A., Suherman, C., Maxiselly, Y., Akbar, M.A., Purwoko, B.A., Prawisudha, P., and Yoshikawa, K. (2019). Liquid feather protein hydrolysate as a potential fertilizer to increase growth and yield of patchouli (Pogostemon cablin Benth) and mung bean (Vigna radiata). International Journal of Recycling of Organic Waste in Agriculture 8, 221-232.
117. Omer, E., Said-Al-Ahl, H., El-Gendy, A., Shaban, K.A., and Hussein, M. (2013). Effect of amino acids application on production, volatile oil and chemical composition of chamomile cultivated in saline soil at sinai. Journal of Applied Sciences Research 9, 3006-3021.
118. Osman, H.S. (2014). Effect of exogenous application of boric acid and seaweed extract on growth, biochemical content and yield of eggplant. Journal of Horticultural Science & Ornamental Plants 6, 133-143.
119. Petoumenou, D.G., and Patris, V.E. (2021). Effects of Several Preharvest Canopy Applications on Yield and Quality of Table Grapes (Vitis vinifera L.) Cv. Crimson Seedless. Plants-Basel 10, 17.
120. Pohl, A., Grabowska, A., Kalisz, A., and Sekara, A. (2019). The Eggplant Yield and Fruit Composition as Affected by Genetic Factor and Biostimulant Application. Notulae Botanicae Horti Agrobotanici Cluj-Napoca 47, 929-938.
121. Popescu, G.C., and Popescu, M. (2018). Yield, berry quality and physiological response of grapevine to foliar humic acid application. Bragantia 77, 273-282.
122. Popko, M., Michalak, I., Wilk, R., Gramza, M., Chojnacka, K., and Gorecki, H. (2018). Effect of the New Plant Growth Biostimulants Based on Amino Acids on Yield and Grain Quality of Winter Wheat. Molecules 23.
123. Pourghasemian, N., Moradi, R., Naghizadeh, M., and Landberg, T. (2020). Mitigating drought stress in sesame by foliar application of salicylic acid, beeswax waste and licorice extract. Agricultural Water Management 231.
124. Pramanick, B., Brahmachari, K., Ghosh, A., and Zodape, S.T. (2016). Effect of seaweed saps derived from two marine algae Kappaphycus and Gracilaria on growth and yield improvement of blackgram. Indian Journal of Geo-Marine Sciences 45, 789-794.
125. Procházka, P., Štranc, P., Pazderů, K., Štranc, J., and Jedličková, M. (2015). The possibilities of increasing the production abilities of soya vegetation by seed treatment with biologically active compounds. Plant, Soil and Environment 61, 279-284.
126. Prokkola, S., and Kivijärvi, P. (2007). Effect of biological sprays on the incidence of grey mould, fruit yield and fruit quality in organic strawberry production. Agricultural and Food Science - AGR FOOD SCI 16, 25-33.
127. Radkowski, A., and Radkowska, I. (2018). Influence of foliar fertilization with amino acid preparations on morphological traits and seed yield of timothy. Plant, Soil and Environment 64, 209-213.
128. Radkowski, A., Radkowska, I., and Godyn, D. (2018). EFFECTS OF FERTILIZATION WITH AN AMINO ACID PREPARATION ON THE DRY MATTER YIELD AND CHEMICAL COMPOSITION OF MEADOW PLANTS. Journal of Elementology 23, 947-958.
129. Rady, M.M., Desoky, E.S.M., Elrys, A.S., and Boghdady, M.S. (2019). Can licorice root extract be used as an effective natural biostimulant for salt-stressed common bean plants? South African Journal of Botany 121, 294-305.
130. Rady, M.M., and Mohamed, G.F. (2015). Modulation of salt stress effects on the growth, physio-chemical attributes and yields of Phaseolus vulgaris L. plants by the combined application of salicylic acid and Moringa oleifera leaf extract. Scientia Horticulturae 193, 105-113.
131. Rahman, M., Mukta, J.A., Sabir, A.A., Gupta, D.R., Mohi-Ud-Din, M., Hasanuzzaman, M., Miah, M.G., Rahman, M., and Islam, M.T. (2018). Chitosan biopolymer promotes yield and stimulates accumulation of antioxidants in strawberry fruit. PloS one 13, e0203769-e0203769.
132. Raposo Junior, J.L., Neto, J.a.G., and Sacramento, L.V.S. (2013). EVALUATION OF DIFFERENT FOLIAR FERTILIZERS ON THE CROP PRODUCTION OF SUGARCANE. Journal of Plant Nutrition 36, 459-469.
133. Rashid, N., Khan, S., Wahid, A., Basra, S.M.A., Alwahibi, M.S., and Jacobsen, S.-E. (2020). Impact of natural and synthetic growth enhancers on the productivity and yield of quinoa (chenopodium quinoa willd.) cultivated under normal and late sown circumstances. Journal of Agronomy and Crop Science n/a.
134. Rathore, S.S., Chaudhary, D.R., Boricha, G.N., Ghosh, A., Bhatt, B.P., Zodape, S.T., and Patolia, J.S. (2009). Effect of seaweed extract on the growth, yield and nutrient uptake of soybean (Glycine max) under rainfed conditions. South African Journal of Botany 75, 351-355.
135. Raverkar, K.P., Pareek, N., Chandra, R., Chauhan, S., Zodape, S.T., and Ghosh, A. (2016). Impact of foliar application of seaweed saps on yield, nodulation and nutritional quality in green gram (Vigna radiata L). Legume Research 39, 315-318.
136. Ronga, D., Caradonia, F., Setti, L., Hagassou, D., Giaretta Azevedo, C.V., Milc, J., Pedrazzi, S., Allesina, G., Arru, L., and Francia, E. (2019). "Effects of innovative biofertilizers on yield of processing tomato cultivated in organic cropping systems in northern Italy", in: Acta Horticulturae.).
137. Rouphael, Y., Giordano, M., Cardarelli, M., Cozzolino, E., Mori, M., Kyriacou, M.C., Bonini, P., and Colla, G. (2018). Plant-and seaweed-based extracts increase yield but differentially modulate nutritional quality of greenhouse spinach through biostimulant action. Agronomy 8.
138. Ruban, S.J., Priya, M.R., Barathan, G., and Kumar, S.M.S. (2019). Effect of foliar application of biostimulants on yield of brinjal (Solanum melongena L.). Plant Archives 19, 2118-2120.
139. Saad-Allah, K.M., Fetouh, M.I., and Elhaak, M.A. (2017). Induction of milk thistle (Silybum marianum L. Gaertn) growth and phytochemicals production by natural stimulants. Journal of Applied Research on Medicinal and Aromatic Plants 6, 101-110.
140. Sabir, A., Yazar, K., Sabir, F., Kara, Z., Yazici, M.A., and Goksu, N. (2014). Vine growth, yield, berry quality attributes and leaf nutrient content of grapevines as influenced by seaweed extract (Ascophyllum nodosum) and nanosize fertilizer pulverizations. Scientia Horticulturae 175, 1-8.
141. Sakr, W.R., El-Sayed, A., Hammouda, A., and El Deen, F.S. (2018). Effect of NPK, aloe gel and moringa extracts on geranium plants. J. Hortic. Sci. Ornam. Plants 10, 01-16.
142. Saruhan, V., Kusvuran, A., and Kokten, K. (2011). The effect of different replications of humic acid fertilization on yield performances of common vetch (Vicia sativa L.). African Journal of Biotechnology 10, 5587-5592.
143. Schoebitz, M., López, M.D., Serri, H., Aravena, V., Zagal, E., and Roldán, A. (2019). Characterization of Bioactive Compounds in Blueberry and Their Impact on Soil Properties in Response to Plant Biostimulants. Communications in Soil Science and Plant Analysis 50, 2482-2494.
144. Selim, E.M., and Ali Mosa, A. (2012). Fertigation of humic substances improves yield and quality of broccoli and nutrient retention in a sandy soil. Journal of Plant Nutrition and Soil Science 175, 273-281.
145. Semida, W.M., Abd El-Mageed, T.A., Hemida, K., and Rady, M.M. (2019). Natural bee-honey based biostimulants confer salt tolerance in onion via modulation of the antioxidant defence system. Journal of Horticultural Science and Biotechnology 94, 632-642.
146. Shah, M.T., Zodape, S.T., Chaudhary, D.R., Eswaran, K., and Chikara, J. (2013). SEAWEED SAP AS AN ALTERNATIVE LIQUID FERTILIZER FOR YIELD AND QUALITY IMPROVEMENT OF WHEAT. Journal of Plant Nutrition 36, 192-200.
147. Shah, S., Hookway, S., Wilkinson, S., and Fletcher, J. (2017). The effect of biostimulants on crop vigour, disease incidence and grain yield of winter wheat and winter oilseed rape. Aspects of Applied Biology, 59-69.
148. Shaheen, A.M., Abd El-Samad, E.H., Rizk, F.A., Behairy, A.G., and Adam, S.M. (2018). EFFECT OF APPLICATION METHODS OF PLANT GROWTH STIMULANTS ON GROWTH AND YIELD OF SNAP BEAN. Journal of Animal and Plant Sciences 28, 854-864.
149. Shaheen, A.M., Ragab, M.E., Rizk, F.A., Mahmoud, S.H., Soliman, M.M., and Omar, N.M. (2017). Effect of some active stimulants on plant growth, tubers yield and nutritional values of potato plants grown in newly reclaimed soil. Research Journal of Pharmaceutical Biological and Chemical Sciences 8, 581-596.
150. Shalaby, T.A., and El-Ramady, H. (2014). Effect of foliar application of bio-stimulants on growth, yield, components, and storability of garlic ('Allium sativum'L.). Australian Journal of Crop Science 8, 271.
151. Sharma, L., Banerjee, M., Malik, G.C., Gopalakrishnan, V.a.K., Zodape, S.T., and Ghosh, A. (2017). Sustainable agro-technology for enhancement of rice production in the red and lateritic soils using seaweed based biostimulants. Journal of Cleaner Production 149, 968-975.
152. Shm, T., Kassim, N., Abourayya, M., and Abdalla, A. (2017). Influence of foliar application with Moringa (Moringa oleifera L.) leaf extract on yield and fruit quality of hollywood plum cultivar. J Hortic 4, 2376-0354.1000193.
153. Soppelsa, S., Kelderer, M., Casera, C., Bassi, M., Robatscher, P., and Andreotti, C. (2018). Use of Biostimulants for Organic Apple Production: Effects on Tree Growth, Yield, and Fruit Quality at Harvest and During Storage. Frontiers in Plant Science 9.
154. Soppelsa, S., Kelderer, M., Testolin, R., Zanotelli, D., and Andreotti, C. (2020). Effect of Biostimulants on Apple Quality at Harvest and After Storage. Agronomy 10, 1214.
155. Spinelli, F., Fiori, G., Noferini, M., Sprocatti, M., and Costa, G. (2009). Perspectives on the use of a seaweed extract to moderate the negative effects of alternate bearing in apple trees. The Journal of Horticultural Science and Biotechnology 84, 131-137.
156. Stamatiadis, S., Evangelou, E., Jamois, F., and Yvin, J.-C. (2021). Targeting Ascophyllum nodosum (L.) Le Jol. extract application at five growth stages of winter wheat. Journal of Applied Phycology, 1-10.
157. Suh, H.Y., Yoo, K.S., and Suh, S.G. (2014). Tuber growth and quality of potato (Solanum tuberosum L.) as affected by foliar or soil application of fulvic and humic acids. Horticulture, Environment, and Biotechnology 55, 183-189.
158. Sulakhudin, Hatta, M., and Suryadi, U.E. (2019). Application of Coastal Sediments and Foliar Seaweed Extract and Its Influence to Soil Properties, Growth and Yield of Shallot in Peatland. Agrivita 41, 450-460.
159. Szczepanek, M. (Year). "Technology of maize with growth stimulants application", in: Engineering for Rural Development), 483-490.
160. Szczepanek, M., Jas´Kiewicz, B., and Kotwica, K. (Year). "Response of barley on seaweed biostimulant application", in: Research for Rural Development), 49-54.
161. Szczepanek, M., Wilczewski, E., Pobereżny, J., Wszelaczyńska, E., and Ochmian, I. (2017). Carrot root size distribution in response to biostimulant application. Acta Agriculturae Scandinavica Section B: Soil and Plant Science 67, 334-339.
162. Szczepanek, M., Wszelaczyńska, E., and Pobereżny, J. (2018). Effect of the seaweed biostimulant application in spring wheat. AgroLife Scientific Journal 7, 131-136.
163. Szot, I., and Wieniarska, J. (2012). Effect of foliar applications of goëmar ® BM 86 and soil applied calcium nitrate on yield and berry quality of two blue honeysuckle cultivars. Acta Scientiarum Polonorum, Hortorum Cultus 11.
164. Szparaga, A., Kocira, S., Kapusta, I., and Zaguła, G. (2021). Prototyping extracts from Artemisia absinthium L. for their biostimulating properties yield-enhancing, and farmer income-increasing properties. Industrial Crops and Products 160, 113125.
165. Tadayyon, A., Beheshti, S., and Pessarakli, M. (2017). Effects of sprayed humic acid, iron, and zinc on quantitative and qualitative characteristics of niger plant (Guizotia abyssinica L.). Journal of Plant Nutrition 40, 1644-1650.
166. Tandon, S., and Dubey, A. (2015). Effects of Biozyme (Ascophyllum nodosum) Biostimulant on Growth and Development of Soybean [Glycine Max (L.) Merill]. Communications in Soil Science and Plant Analysis 46, 861-874.
167. Tarantino, A., Lops, F., Disciglio, G., and Lopriore, G. (2018). Effects of plant biostimulants on fruit set, growth, yield and fruit quality attributes of ‘Orange rubis®’apricot (Prunus armeniaca L.) cultivar in two consecutive years. Scientia Horticulturae 239, 26-34.
168. Taskos, D., Stamatiadis, S., Yvin, J.C., and Jamois, F. (2019). Effects of an Ascophyllum nodosum (L.) Le Jol. extract on grapevine yield and berry composition of a Merlot vineyard. Scientia Horticulturae 250, 27-32.
169. Tejada, M., Rodríguez-Morgado, B., Paneque, P., and Parrado, J. (2018). Effects of foliar fertilization of a biostimulant obtained from chicken feathers on maize yield. European Journal of Agronomy 96, 54-59.
170. Telekalo, N., and Melnyk, M. (2020). Agroecological substantiation of medicago sativa cultivation technology. Agronomy Research 18, 2613-2626.
171. Temiz, M., Karahan, E., and Koca, Y.K. (2009). Effects of Humic Substances on Cotton (Gossypium hirsutum L.). Asian Journal of Chemistry 21, 1983-1989.
172. Trivedi, K., Anand, K.G.V., Kubavat, D., Kumar, R., Vaghela, P., and Ghosh, A. (2017). Crop stage selection is vital to elicit optimal response of maize to seaweed bio-stimulant application. Journal of Applied Phycology 29, 2135-2144.
173. Wadas, W., and Dziugieł, T. (2020). Changes in assimilation area and chlorophyll content of very early potato (Solanum tuberosum L.) cultivars as influenced by biostimulants. Agronomy 10.
174. Wang, M., Chen, Y., Zhang, R., Wang, W., Zhao, X., Du, Y., and Yin, H. (2015). Effects of chitosan oligosaccharides on the yield components and production quality of different wheat cultivars (Triticum aestivum L.) in Northwest China. Field Crops Research 172, 11-20.
175. Wilczewski, E., Szczepanek, M., and Wenda-Piesik, A. (2018). Response of sugar beet to humic substances and foliar fertilization with potassium. Journal of Central European Agriculture 19, 153-165.
176. Wolski, K., Biernacik, M., Świerszcz, S., Talar-Krasa, M., and Leshchenko, O. (2019). Effect of the application of a biostimulant and mineral fertilizers on the concentration of mineral elements in the sward of forage mixtures cultivated on light soil. Journal of Elementology 24, 385-397.
177. Yasmeen, A., Arif, M., Hussain, N., Malik, W., and Qadir, I. (2016). Morphological, Growth and Yield Response of Cotton to Exogenous Application of Natural Growth Promoter and Synthetic Growth Retardant. International Journal of Agriculture and Biology 18, 1109-1121.
178. Younes, N.A., Rahman, M.M., Wardany, A.A., Dawood, M.F.A., Mostofa, M.G., Keya, S.S., Latef, A., and Tran, L.S.P. (2021). Antioxidants and Bioactive Compounds in Licorice Root Extract Potentially Contribute to Improving Growth, Bulb Quality and Yield of Onion (Allium cepa). Molecules 26, 16.
179. Zarzecka, K., Gugała, M., Sikorska, A., Grzywacz, K., and Niewęgłowski, M. (2020). Marketable yield of potato and its quantitative parameters after application of herbicides and biostimulants. Agriculture (Switzerland) 10.
180. Zodape, S., Kawarkhe, V., Patolia, J., and Warade, A. (2008). Effect of liquid seaweed fertilizer on yield and quality of okra (Abelmoschus esculentus L.). Journal of Scientific and Industrial Research.
181. Zodape, S.T., Gupta, A., Bhandari, S.C., Rawat, U.S., Chaudhary, D.R., Eswaran, K., and Chikara, J. (2011). Foliar application of seaweed sap as biostimulant for enhancement of yield and quality of tomato (Lycopersicon esculentum Mill.). Journal of Scientific & Industrial Research 70, 215-219.
