# Supplementary material for: A Meta-Analysis of Biostimulant Yield Effectiveness in Field Trials
Source: Front Plant Sci. 2022 Apr 14;13:836702. doi: 10.3389/fpls.2022.836702 (PMC9047501; doi:10.3389/fpls.2022.836702)
Supplement: Supplementary file 4 [file Data_Sheet_3.docx]

Supplementary Material

# Supplementary Data

**Supplementary Data 1.** Studies included for meta-analysis.

**Supplementary Data 2.** Dataset collected for meta-analysis.

# Supplementary Figures and Tables

## Supplementary Figures

**Supplementary Figure 1.** PRISMA 2020 flow diagram.

It was provided as a separate file.


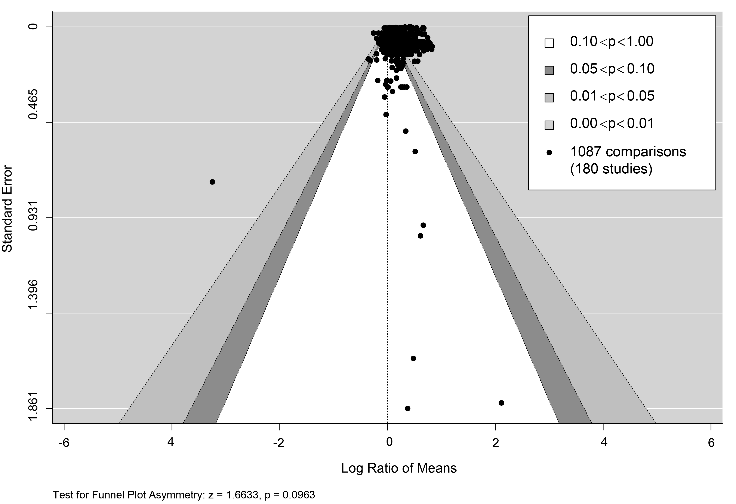


**Supplementary Figure 2.** Contour-enhanced funnel plot for publication bias based on the standard errors in the random-effect models of 187 studies used in this meta-analysis. Each point represents the standard error located in distinct levels of confidence limits for a single study. Asymmetry analysis of Egger's test is indicated at the bottom left, where *p* > 0.001 means there is no evidence for asymmetry.

## Supplementary Figures and Tables

**Supplementary Table 1.** The biostimulant categories used in this meta-analysis according to the substance resources, adapted from (Du Jardin, 2015).

| **Biostimulant Category** | **Natural resources/substances** | **Bioactive compounds** | **Reference** |
| --- | --- | --- | --- |
| Chitosan (Chi) | Shrimp or crab shells | Co-polymers of N-acetyl-d-glucosamine and d-glucosamine | Reviewed in (Pichyangkura and Chadchawan, 2015) |
| Humic and fulvic acids (HFA) | plant and animal matter, and microbial metabolism | humic acids and fulvic acids | Reviewed in (Canellas et al., 2015) |
| Protein hydrolysates (PHs) | Both animal and plant biomass | peptides and free amino acids | Reviewed in (Colla et al., 2015) |
| Phosphites (Phi) | P-containing nutrients | phosphite (Phi; H_2_PO_3_^−^) or its conjugate phosphorous acid (H_3_PO_3_) | Reviewed in (Gómez-Merino and Trejo-Téllez, 2015) |
| Seaweed extracts (SWE) | Red, green, and brown macroalgae species | Primary metabolites, i.e., carbohydrates, proteins, and amino acids | Reviewed in (Chiaiese et al., 2018) |
| Silicon (Si) | biogenic silica soils | monomeric silicic acid (H_4_SiO_4_) | Reviewed in (Savvas and Ntatsi, 2015) |
| Plant extract (PE) | Plant tissues except for seaweed and others aiming for HFA and PHs | Various plant metabolites | Reviewed in (Ali et al., 2020) |

# Reference

Ali, Q., Shehzad, F., Waseem, M., Shahid, S., Hussain, A.I., Haider, M.Z., Habib, N., Hussain, S.M., Javed, M.T., and Perveen, R. (2020). "Plant-Based Biostimulants and Plant Stress Responses," in *Plant Ecophysiology and Adaptation under Climate Change: Mechanisms and Perspectives I: General Consequences and Plant Responses,* ed. M. Hasanuzzaman. (Singapore: Springer Singapore), 625-661.

Canellas, L.P., Olivares, F.L., Aguiar, N.O., Jones, D.L., Nebbioso, A., Mazzei, P., and Piccolo, A. (2015). Humic and fulvic acids as biostimulants in horticulture. *Scientia Horticulturae* 196**,** 15-27.

Chiaiese, P., Corrado, G., Colla, G., Kyriacou, M.C., and Rouphael, Y. (2018). Renewable sources of plant biostimulation: microalgae as a sustainable means to improve crop performance. *Frontiers in plant science* 9**,** 1782.

Colla, G., Nardi, S., Cardarelli, M., Ertani, A., Lucini, L., Canaguier, R., and Rouphael, Y. (2015). Protein hydrolysates as biostimulants in horticulture. *Scientia Horticulturae* 196**,** 28-38.

Du Jardin, P. (2015). Plant biostimulants: definition, concept, main categories and regulation. *Scientia Horticulturae* 196**,** 3-14.

Gómez-Merino, F.C., and Trejo-Téllez, L.I. (2015). Biostimulant activity of phosphite in horticulture. *Scientia Horticulturae* 196**,** 82-90.

Pichyangkura, R., and Chadchawan, S. (2015). Biostimulant activity of chitosan in horticulture. *Scientia Horticulturae* 196**,** 49-65.

Savvas, D., and Ntatsi, G. (2015). Biostimulant activity of silicon in horticulture. *Scientia Horticulturae* 196**,** 66-81.
